# Dose-response of tomato fruit yield to far-red fraction in supplementary lighting

**DOI:** 10.3389/fpls.2025.1618171

**Published:** 2025-07-10

**Authors:** Elena Vincenzi, Aron Moehn, Emmanouil Katsadas, Sana Karbor, Esther de Beer, Frank Millenaar, Leo F M Marcelis, Ep Heuvelink

**Affiliations:** ^1^ Horticulture and Product Physiology, Department of Plant Science, Wageningen University and Research, Wageningen, Netherlands; ^2^ Signify Netherlands B.V., Eindhoven, Netherlands; ^3^ BASF–Nunhems, Nunhem, Netherlands

**Keywords:** tomato, far-red light, radiation use efficiency, electricity use efficiency, fruit quality, vertical light distribution, photosynthesis, yield component analysis

## Abstract

Supplementary LED lighting in greenhouse horticulture is typically rich in red light (R; 600–700 nm), while it lacks far-red light (FR; 700–800 nm), resulting in growing conditions with lower-than-solar far-red fractions [<0.46; FR/(R + FR)]. In these light environments, the addition of FR can improve tomato harvest index and fruit yield (ripe fruit fresh weight). While fruit yield increases linearly with the dose of FR at low FR fractions (0.1–0.28), it is unknown whether this relationship holds at higher FR levels, up to and above solar FR fractions. In this study, the relationship between tomato fruit yield and the FR fraction in supplementary lighting was quantified. Two cluster tomato cultivars ‘Foundation’ and ‘Trevine’ were grown in two greenhouse compartments for 20 weeks during the winter season (September to February). Different fractions of supplementary FR (0.22 to 0.49) were applied while maintaining a constant supplementary photosynthetic photon flux density of 250 µmol m^−2^ s^−1^ and 16-hour photoperiod. A yield component analysis was used to identify the key physiological drivers of the FR effect on yield. Additionally, fruit quality at harvest (total soluble solids, soluble sugars, and pH) and shelf-life were assessed. Additional FR increased fruit yield up to an FR fraction of 0.40, where the highest effect was recorded (+16% fruit yield for both cultivars). Fruit yield increases under additional FR were mostly associated with increased plant dry weight, with a small yet significant increase in the fraction of dry matter partitioned to the fruits. The radiation use efficiency (g fruit fresh weight mol^−1^) and electricity use efficiency of supplementary lighting (g fruit fresh weight kWh^−1^) decreased at higher FR fractions (0.44 and 0.49). Finally, additional FR had a minimal effect on fruit quality and shelf-life. We conclude that adding FR to supplementary lighting can increase tomato fruit yield linearly up to an FR fraction of 0.40, while at higher FR fractions, further increases in FR have limited or even negative effects on yield and decrease radiation and electricity use efficiency.

## 1 Introduction

Tomato (*Solanum lycopersicum* L.) is one of the most widely grown horticultural crops worldwide as well as a model species for studying fruit development. Tomato cultivation in northern countries is predominately carried out under high-tech greenhouses, where climate control technologies such as heating and supplementary lighting are required to enable year-round fruit production (Pinho and Halonen, 2017; Ahamed et al., 2019). The development of light-emitting diodes (LEDs) has strongly advanced research on the light spectrum regulation of crop growth and development (Heuvelink et al., 2024). The adoption of LEDs for supplementary lighting has created new growing environments presenting distinct characteristics, limitations, and opportunities (van Delden et al., 2021).

Currently, supplementary lighting contains high fractions of red light (R, 600–700 nm), while mostly lacking far-red light (FR; 700–800 nm). This choice reflects the higher photosynthetic efficiency and leaf absorption of red light (McCree, 1971), as well as the high efficacy of red LEDs (Kusuma et al., 2020). The resulting spectra present FR fractions [FR/(R + FR); Kusuma and Bugbee, 2021] as low as 0–0.1, a novel condition for plants, compared to FR fractions of approximately 0.46 under direct solar radiation at solar noon [phytochrome photostationary state (PSS) ~ 0.70, R/FR ~ 1.2] and higher values under canopy shading (Cummings et al., 2007; Kalaitzoglou et al., 2019). FR has been shown to regulate plant growth and development by mediating photomorphogenic responses (Kami et al., 2010) as well as by increasing the photochemical efficiency of shorter-wavelength radiation, resulting in improved photosynthesis (Emerson and Rabinowitch, 1960; Hogewoning et al., 2012; Zhen and Bugbee, 2020). It has been suggested that the absence of FR in the growing environment may limit crop performance (Kalaitzoglou et al., 2019), and research has focussed on incorporating FR into red-dominated light recipes (Demotes-Mainard et al., 2016; Paradiso and Proietti, 2022). In greenhouse compartments with low solar radiation, supplementary LED lighting with additional FR can increase fruit yield and the fraction of dry matter partitioned to the fruits in tomato plants (Ji et al., 2019, 2020; Kalaitzoglou et al., 2019; Kim et al., 2019, 2020; Vincenzi et al., 2024). In these environments, higher fruit yield results from increased individual fruit fresh weight and is accompanied by enhanced starch and sugar metabolism within the fruits and higher fruit dry matter content (Fanwoua et al., 2019; Ji et al., 2020; Kim et al., 2020). Additional FR has also been linked to changes in the gene expression of fruit sugar transporters and enhanced fruit sink strength (Ji et al., 2020; Vincenzi et al., 2024), in some cases leading to increased total soluble solids content (°Brix) and improved fruit quality at harvest (Kim et al., 2020). Interestingly, recent studies have reported that the yield-promoting effect of FR was linearly correlated with both its intensity and the duration of daily application across multiple tomato cultivars (Ji et al., 2019; Vincenzi et al., 2024). However, these correlations have been observed within a limited range of FR fractions (0–0.1 to 0.26–0.28), and no study has yet quantitatively assessed fruit yield variation as a dose response to higher FR fractions. Quantifying the fruit yield response at higher FR fractions is critical for determining whether yield continues to increase proportionally with additional FR input. Such insights are essential for optimising FR application and maximising yield outcomes while minimising energy consumption and production costs.

In this study, we aimed to quantify the relationship between the FR fraction in supplementary lighting and tomato fruit yield, within a range of FR supplementation spanning from relatively low values (0.22) to above direct solar radiation levels (0.49). We hypothesised that, while fruit yield would increase linearly at lower FR fractions, this trend would not persist at higher fractions, where yield would instead reach an optimum or saturation point. To test this hypothesis, we imposed a gradient of FR supplementary lighting by keeping the photosynthetic photon flux density stable and increasing the intensity of additional FR. We grew tomato plants for 20 weeks to determine fruit yield, fruit quality at harvest, and fruit shelf-life. Finally, we performed a yield component analysis to determine the relative contribution of the physiological and morphological components underlying the FR effect on fruit yield (Higashide and Heuvelink, 2009; Ji et al., 2019; Vincenzi et al., 2024).

## 2 Materials and methods

### 2.1 Plant material and growth conditions

On September 13, 2022, uniform plants of the commercial cluster tomato hybrids, *S. lycopersicum* L. cv. Foundation and cv. Trevine (BASF—Nunhems, Nunhem, the Netherlands), were transplanted into two adjacent greenhouse compartments at Wageningen University and Research (52°N, 6°E, Wageningen, the Netherlands). Plants were approximately 50 cm tall, with the first truss having not yet reached anthesis. The plants were placed on 100 × 15 × 7.5-cm stonewool slabs (Grodan, Roermond, the Netherlands) at a planting density of 2.7 plants per m^2^ and grown according to high-wire tomato cultivation practices. When the plants reached full canopy height (3 m), they were lowered every week to ensure a minimum distance of 50 cm between the canopy top and the lamps. Every week, the three oldest leaves at the bottom of each plant and all side shoots were removed. The first flowering truss was pruned to five flowers, while subsequent trusses for both varieties were pruned to six flowers, following standard practices for these cultivars. Pollination was facilitated by bumblebees (Natupol Smart, Koppert, Berkel en Rodenrijs, the Netherlands).

Plants were drip irrigated with a standard nutrient solution for tomato growth containing 1.2 mM NH_4_^+^, 11.0 mM K^+^, 6.3 mM Ca^2+^, 2.8 mM Mg^2+^, 18.4 mM NO_3_^−^, 5.1 mM SO_4_^2−^, 1.7 µM PO_4_^2−^, 25.0 µM Fe^3+^, 10.0 µM Mn^2+^, 5.0 µM Zn^2+^, 30.0 µM H_2_BO_3_^−^, 0.8 µM Cu^2+^, and 0.5 µM MoO_4_^2−^ (electric conductivity 2.9 dS m^−1^ and pH 6.0). The average air temperature was 21.9°C ± 0.6°C during the day and 18.8°C ± 0.2°C during the night, with a daily relative humidity of 76% ± 4%. CO_2_ enrichment started 5 weeks after transplant, when tomato plants are strongly source limited (Li et al., 2015); the CO_2_ concentration was kept at an average of 530 ± 35 µmol mol^−1^ until the end of the experiment.

### 2.2 Supplementary FR treatments

A gradient of supplementary FR was realised by progressively increasing the FR light intensity from one side to the other within a greenhouse compartment. Six distinct FR treatments were established along the gradient (**Table 1**), each corresponding to a double plant row. One border row was present on each side of the FR gradient. All treatments received supplementary white (W) lighting at 248 ± 3 µmol m^−2^ s^−1^, provided by LED modules (GreenPower LED TLL 630 DRW, Spectrum VSN2, Philips, Eindhoven, the Netherlands) with an R:G:B ratio of 45:35:20. The W light spectrum contained less red light than it is typically used in tomato cultivation, and it was chosen to achieve high FR fractions [FR/(R + FR)] in our light treatments. FR was provided by LED modules (GreenPower LED2.2 FR 150 RO, Philips), with the number of FR modules per unit area increasing along the gradient to achieve higher FR intensities. To compensate for the additional shading caused by the FR modules on one side of the gradient, wooden dummies were evenly installed across the entire gradient (**Supplementary Figure S1**). The photoperiod of all supplementary lighting was set to 13 hours of light per day upon transplant and then gradually increased to 16 hours of light per day at 59 days after transplant (DAT). From 59 DAT until the end of the experiment, all supplementary lighting was turned on around midnight and remained on until sunset at approximately 4 pm (**Supplementary Table S1**). The intensity and spectral distribution of the LED lighting in the Photosynthetically Active Radiation (PAR) and FR spectra were measured at 2 m above the ground, before transplanting, using a spectrometer (Li-180, LI-COR Biosciences, Lincoln, NE, USA; **Supplementary Figure S2**). The blue, green, red, and far-red spectra supplied by LED modules in this experiment peaked at 448, 564, 665, and 738 nm, respectively. Solar radiation accounted for 15% of the total (solar + supplementary lighting) daily light integral of PAR radiation on average during the growth period (**Supplementary Figure S3**). Solar radiation increased the total (solar + supplementary lighting) FR fraction experienced by the plants in the lowest FR treatments (FR_0.22_ and FR_0.29_), while its effect was minimal on the other four treatments (**Table 1**).

**Table 1:** Photosynthetic photon flux density (PPFD; 401–700 nm), photon flux density (PFD) of red (PFD-Red; 601–700 nm), PFD of far-red (PFD-FR; 701–800 nm), and FR fraction of the light treatments applied in this experiment (Supplementary lighting).

Light treatments	PPFD	PFD-FR	PFD-Red	FR fraction	FR fraction
	Supplementary lighting				Solar + supplementary lighting
	(μmol s^−1^ m^−2^)	(μmol s^−1^ m^−2^)	(μmol s^−1^ m^−2^)	FR/(R + FR)	FR/(R + FR)
FR_0.22_	237 ± 3	28 ± 1	101 ± 4	0.22 ± 0.006	0.26 ± 0.003
FR_0.29_	253 ± 2	45 ± 1	109 ± 3	0.29 ± 0.005	0.32 ± 0.002
FR_0.35_	251 ± 2	59 ± 1	109 ± 3	0.35 ± 0.006	0.37 ± 0.001
FR_0.40_	254 ± 2	73 ± 2	111 ± 2	0.40 ± 0.008	0.41 ± 0.001
FR_0.44_	254 ± 3	89 ± 3	111 ± 3	0.44 ± 0.009	0.44 ± 0.0002
FR_0.49_	243 ± 3	103 ± 3	107 ± 3	0.49 ± 0.01	0.49 ± 0.0004

The black-out screen on top of the greenhouse was closed during the measurements of the light treatments to block incoming solar radiation. Values represent mean ± SEM (n = 4, total number). FR fraction of Solar + Supplementary lighting was calculated per light treatment as a daily average of hourly values. Values represent average daily FR fraction ± SEM (n = 140, total number of days in the experiment).

Within each greenhouse compartment, two FR gradients were realised, separated by a 1-m-long double-layer white plastic sheet positioned at the height of the lamps. The two gradients within the same compartment were oriented in opposite directions (**Supplementary Figure S4**). Airflow was facilitated by a vertical fan located in the centre of each compartment above the canopy.

### 2.3 Plant measurements

#### 2.3.1 Growth and development parameters

Growth and development parameters were determined on six plants per experimental unit (plant row with a specific FR treatment in the gradient). Stem length, leaf number, flowering rate, and fruit ripening rate were measured weekly until 56 DAT and thereafter every 2 weeks until the end of the experiment. Fruit trusses were harvested when the most distal fruit reached the “turning stage” and all the other fruits reached at least the “light red stage” (USDA, 1991). Fruit harvest was carried out twice per week from 63 DAT until the end of the experiment, recording fruit fresh weight and fruit number. The leaf fresh weight removed during the weekly pruning was recorded. To estimate leaf and fruit dry matter contents, the fresh and dry weights of a sample of five pruned leaves and three ripe fruits were measured every 2 weeks.

Fruit sink strength was estimated based on fruit growth under non-limiting assimilate supply (potential fruit growth, Marcelis, 1996) for only three FR treatments (FR_0.22_, FR_0.40_, and FR_0.49_) due to time constraints. For this measurement, three plants per experimental unit were pruned to one fruit per truss. This truss pruning protocol was applied on all flowering trusses starting from the anthesis of the third truss onwards. The first two trusses were allowed to develop a standard number of flowers until the anthesis of the third truss to support more balanced plant growth during the first weeks after transplant. Consequently, data from trusses 1 and 2 were excluded from the analysis. Ripe fruits were harvested individually twice per week, and their fresh weight was recorded. Dry matter content was determined every 2 weeks.

#### 2.3.2 Radiation and electricity use efficiency

The ripe fruit fresh weight produced per unit of incident photon flux density (PFD) and kWh of electricity from supplementary lighting was calculated to assess the efficiency of the supplementary lighting treatments for fruit production. Incident PFD represented the sum of supplementary PFD and solar PFD. Supplementary PFD was calculated based on the intensity of supplementary lighting and the number of lighting hours. Solar PFD was determined from solar radiation data collected by a solarimeter outside the greenhouse compartment, adjusted for the greenhouse transmissivity measured under the experimental setup (0.26), which included extensive woodwork to support the lighting modules and the spectral photon distribution of solar radiation (ASTM G173-03). The electricity consumption of supplementary lighting was estimated using 2.5 µmol J^−1^ and 3.6 µmol J^−1^ as photon efficacy values of white and far-red LEDs, respectively. To assess the effects of additional FR lighting under constant PFD, the radiation and electricity use efficiency that could be obtained were estimated by replacing the additional FR in each light treatment with an equivalent amount of PAR. The lowest FR treatment (FR_0.22_) was used as a baseline for these simulations. The simulated fruit yield under increased PAR was based on the relationship between tomato fruit fresh weight and PAR light integral, which indicates an average 0.85% increase in fruit yield for every 1% increase in PAR light integral (Marcelis et al., 2006).

#### 2.3.3 Fruit quality at harvest

Fruit quality at harvest was assessed by total soluble solids content (°Brix), pH, and soluble sugar content in ripe fruits. Total soluble solids content and pH were measured six times during the experiment, approximately once every 2 weeks, while soluble sugar measurements were carried out three times (98, 119, and 133 DAT). Total soluble solids content and pH measurements were carried out on three fruits per experimental unit, and soluble sugar measurements were carried out on three pooled samples per experimental unit, each sample consisting of six fruits. Fruits were randomly selected from different plants, ensuring that only the second or third proximal fruits from each truss were used. Fruits for soluble sugar measurements were consistently harvested between 9 and 12 am. Total soluble solids content and pH were measured from a 7-mL sample of tomato juice using a digital refractometer (RF 232, Euromex, Arnhem, the Netherlands) and a pH meter (HI2210, Hanna Instruments, Nieuwegein, the Netherlands), respectively. The fruits selected for soluble sugar quantification were cut into eight regular wedges, and half a wedge per fruit was randomly selected, frozen in liquid nitrogen, freeze-dried (Alpha 1–4 LSCbasic, Salm en Kipp, Breukelen, the Netherlands), and ground using mortar and pestle. Glucose, fructose, and sucrose concentrations were quantified as described by Ji et al. (2020), with the same equipment and minor adjustments: 15 mg of the pooled freeze-dried samples was weighted and used for the extraction, and samples were diluted 50-fold with MilliQ water before quantification (Full method: **Supplementary Method S1**).

#### 2.3.4 Shelf-life

Fruit shelf-life was determined by monitoring the decline in fruit quality from harvest until it reached a level of reduced marketability, which marked the end of the measurement. The shelf-life measurement was conducted twice. Each measurement included three FR treatments (FR_0.22_, FR_0.40_, and FR_0.49_), with each treatment represented by three replicates per experimental unit. Each replicate consisted of three tomato fruits stored together in the same closed plastic box (18 × 16 × 6.5 cm) with holes to allow air circulation (**Supplementary Figure S5**). Tomato fruits for the shelf-life experiments were harvested at the “light-red stage” (USDA, 1991) and stored, without removing the calyx and the pedicel, at high relative humidity, at 20°C, in the dark for the entire duration of the measurement. Three times per week, the fruit quality index of each fruit was qualitatively assessed based on the average of three parameters: colour, firmness, and shape. The scoring system for these three parameters was adapted from Kader et al. (1973) (**Supplementary Table S2**). At the end of the experiment, the decline in the fruit quality index over time was analysed using a linear regression model, and the slope of the regression was determined for each experimental unit. Based on the regression slope, shelf-life was determined as the number of days required for the fruit quality index to reach the threshold of reduced marketability.

#### 2.3.5 Leaf photosynthesis

Leaf photosynthesis was measured under prevailing light conditions using a portable photosynthesis system (LI-6400XT, LI-COR Biosciences, Lincoln, NE, USA) between 128 and 134 DAT. Measurements were taken on five plants per experimental unit using a transparent 6-cm^2^ leaf chamber. The conditions inside the leaf chamber were set to 23°C temperature, 65% relative humidity, 500 µmol s^−1^ airflow, and 400 µmol mol^−1^ CO_2_. To minimise the solar radiation effect on leaf photosynthesis, measurements were conducted on cloudy days, and leaves were measured without altering their position. The fourth or fifth leaf from the apex (length ≥ 5 cm) was used for measurement. After checking the stability of stomatal conductance and instantaneous photosynthesis, the average photosynthesis rate over a 15-second interval was recorded. The relative photosynthesis rate was calculated by normalising instantaneous photosynthesis against the incident photosynthetic photon flux density (PPFD), recorded just before each measurement using a spectrometer (LI-180, LI-COR Biosciences) held below the leaf chamber glass. Incident PPFD inside the leaf chamber was within the linear part of the light response curve for all measurement points (PPFD in the range of 150 to 340 µmol m^−2^ s^−1^, with 95% of the data below 300 µmol m^−2^ s^−1^).

#### 2.3.6 Plant destructive harvest

Destructive measurements were conducted on six plants at 140–143 DAT (final harvest). Recorded parameters included stem length, leaf number (length ≥ 2 cm), truss number (with at least one open flower), and fruit number (fruit diameter ≥ 2 cm). The total leaf area was measured using a leaf area meter (LI-3100C area meter, LI-COR Biosciences). After drying to a constant weight, the dry weights of stems, leaves, and fruits were determined per plant (ventilated oven, 75 hours, 105°C). Plant dry weight combined the dry weight from the final harvest and the cumulative dry weight of ripe tomatoes harvested during the experiment and pruned leaves.

#### 2.3.7 Vertical light profile and light extinction coefficient

The light extinction coefficient of the tomato canopy was determined for only three FR treatments (FR_0.22_, FR_0.40_, and FR_0.49_) at 49 DAT and 120 DAT. PFD was measured every 30 cm from the top of the canopy (above the apex) to the bottom (below the last leaf) using a spectrometer (LI-180, LI-COR Biosciences). The fraction of light remaining at each height (vertical light profile) was plotted separately for PAR and FR. The extinction coefficient was calculated for PAR only, according to Higashide and Heuvelink (2009). The black-out screen on top of the greenhouse was closed during the light measurements to block incoming solar radiation. Data from the first (49 DAT) and second (120 DAT) vertical light profiles presented the same trend and were averaged together for the data analysis.

### 2.4 Yield component analysis

Differences in fruit yield (ripe fruit fresh weight) were analysed using a yield component analysis as described by Vincenzi et al. (2024) (**Supplementary Figure S6**). In short, ripe fruit fresh weight was dissected into two components, fruit dry matter content and total fruit dry weight. Total fruit dry weight included both harvested ripe fruits and those remaining on the plant at the time of final harvest, and it is the product of plant dry weight and the fraction of dry matter partitioned to the fruits. The fraction to fruit was further broken down into the total number of fruits per plant and potential fruit weight, with total fruit number determined by the number of fruits per truss and truss appearance rate. Plant dry weight was separated into fractions of PAR light intercepted by the canopy and its efficiency in converting intercepted PAR light into dry weight (light use efficiency), both of which were influenced by the PAR light extinction coefficient. Light use efficiency was determined as plant dry weight per unit of solar and supplemental PPFD. Finally, light use efficiency was affected by the relative leaf photosynthesis rate, while the fraction of PAR light intercepted by the canopy depended on the leaf area index, which in turn was determined by the leaf number and leaf area per leaf.

### 2.5 Experimental design and statistical analysis

In one greenhouse compartment, cv. Foundation was grown, and in the other compartment, cv. Trevine. Each greenhouse compartment was divided into two halves, with an FR gradient for each half. Within each FR gradient, six FR treatments were established, each corresponding to an entire double row of tomato plants. Each plant row within an FR gradient, assigned to a specific FR treatment, served as the experimental unit in our setup. Data were analysed using a split-plot ANOVA, with cultivar as the main factor, the FR fraction as the split factor, and the greenhouse half as the blocking factor. The FR fraction was analysed as a quantitative factor. Statistical analyses were performed using GENSTAT (22nd edition, VSN International, London, UK). Outliers, defined as values exceeding 1.5 times the interquartile range from the first and third quartiles, were excluded from the analysis (<3% of data excluded). Averages and standard errors of the mean (SEMs) were calculated based on two experimental units, with each experimental unit representing the mean of six plants, unless stated otherwise. Due to the lower statistical power of our experimental design, a significance level of 0.1 instead of the more common 0.05 was used, aligning with the approach taken by Vincenzi et al. (2024). The normal distribution of the residuals was checked and confirmed using the Shapiro–Wilk test at p = 0.05, while homogeneity of variance was assumed. Fisher’s protected Least Significant Difference (LSD) test was used to assess differences between treatments.

## 3 Results

### 3.1 Fruit production

**Figure 1:**
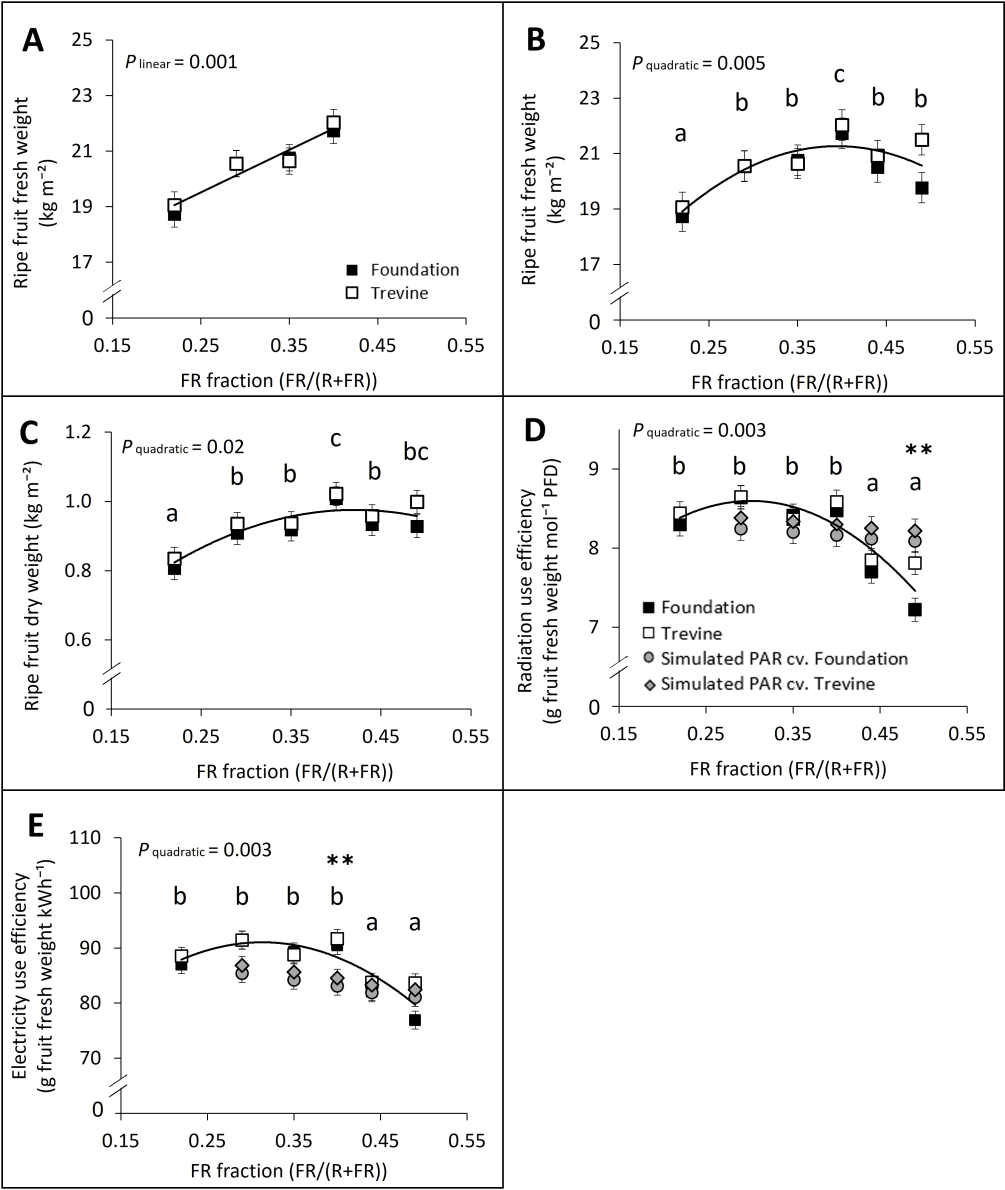
Effects of FR fraction in supplementary light on fruit yield (ripe fruit fresh weight; **A, B**), ripe fruit dry weight **(C)**, radiation use efficiency **(D)**, and electricity use efficiency **(E)** for cv. Foundation and cv. Trevine. Trendlines are depicted to show a significant linear or quadratic relationship with the FR fraction (p < 0.1, averaged over both cultivars). For significant quadratic relationships, letters denote significant differences between treatments, as determined by Fisher’s protected LSD test. The radiation and electricity use efficiency were calculated as fruit yield per unit of PFD and kWh, respectively. Grey markers represent the simulated radiation and electricity use efficiency expected if the additional FR in each light treatment was replaced by PAR, based on Marcelis et al. (2006), for cv. Foundation (round marker) and cv. Trevine (diamond marker). The lowest FR treatment (FR_0.22_) was used as baseline for the PAR simulation. Asterisks indicate a significant difference between radiation and electricity use efficiency measured and simulated for a specific FR treatment as determined by Fisher’s protected LSD test (*p < 0.1; **p < 0.05). Each data point represents the average of two experimental units ± SEM, where the value per experimental unit is the average of six plants. The data refer to cumulative values over a period of 10 weeks, from the first to last fruit harvest, 63 to 139 DAT. FR, far-red light; PFD, photon flux density; DAT, days after transplant.

We assessed the fruit yield of tomato plants grown under a gradient of supplementary FR lighting, with the FR fraction ranging from 0.22 to 0.49, where the maximum is just above the value of direct solar radiation (FR fraction ~ 0.46, R/FR ~ 1.2). Fruit yield increased linearly with the FR fraction in supplementary light up to FR_0.40_, where the highest values were recorded (**Figure 1A**). At FR_0.40_, fruit yield was 16% higher than at FR_0.22_ (lowest FR) and 6% higher than at FR_0.49_ (highest FR) when averaged across the two cultivars (**Figure 1B**). The decrease in fruit yield beyond FR_0.40_ was more pronounced for cv. Foundation than for cv. Trevine (−9% and −2% FR_0.49_ versus FR_0.40_, respectively). Ripe fruit dry weight followed a similar trend (**Figure 1C**), increasing up to FR_0.40_, but remained stable thereafter, with no significant difference between FR_0.40_ and FR_0.49_ (p = 0.165). Radiation use efficiency (RUE) remained stable from FR_0.22_ to FR_0.40_, with an average value of 7.98 g of ripe fruit fresh weight produced per mol of total (solar + supplementary lighting) PFD (**Figure 1D**). However, increasing the FR fraction further, from 0.40 to 0.49, led to a 10%–18% decrease in RUE for cv. Trevine and cv. Foundation, respectively. We compared these results, obtained by increasing PFD with additional FR, to a RUE simulation where PFD was increased by additional PAR, instead of FR. For this simulation, we used the relationship between tomato fruit fresh weight and PAR integral reported by Marcelis et al. (2006), which indicates an average increase in fruit fresh weight of 0.85% per 1% increase in PAR light integral (**Figure 1D**, grey markers). The RUE values recorded for FR_0.29_ to FR_0.44_ were not significantly different from the values obtained from the PAR simulation. Electricity use efficiency of supplementary lighting (EUE) followed a similar trend as RUE across the gradient of FR fractions (**Figure 1E**). EUE values for FR_0.29_, FR_0.35_, and FR_0.40_ were higher than the values obtained by the PAR simulation (**Figure 1E**, grey markers).

### 3.2 Fraction of dry matter partitioned to the fruits, flowering, and fruit ripening rate

**Figure 2:**
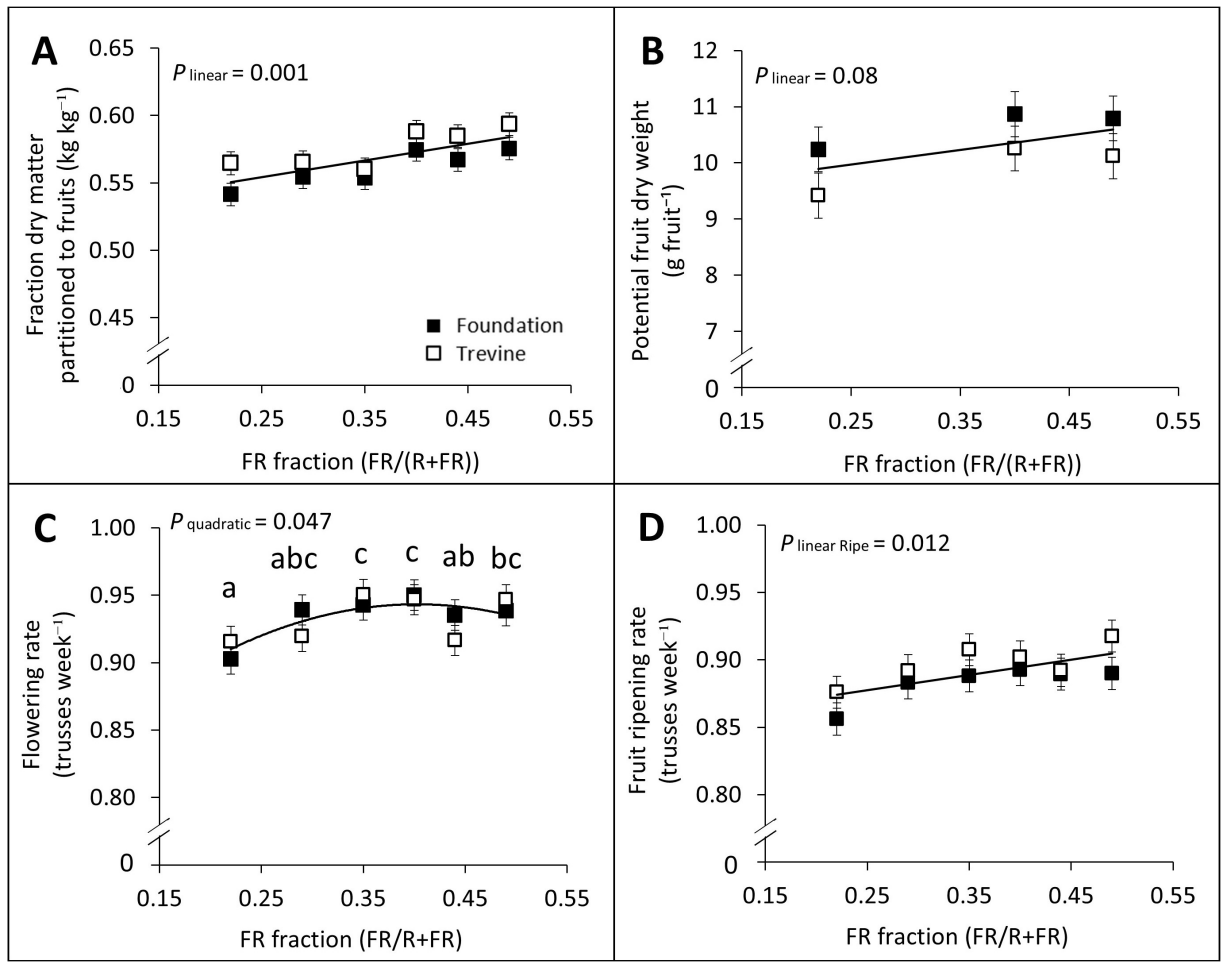
Effects of FR fraction in supplementary light on fraction of dry matter partitioned to the fruits **(A)**, potential fruit dry weight **(B)**, flowering rate **(C)**, and fruit ripening rate **(D)** for cv. Foundation and cv. Trevine. Trendlines are depicted to show a significant linear or quadratic relationship with the FR fraction (p < 0.1, averaged over both cultivars). For significant quadratic relationships, letters denote significant differences between treatments, as determined by Fisher’s protected LSD test. Measurements of potential fruit dry weight required a specific pruning protocol that was carried out only for three FR treatments (FR_0.22_, FR_0.40_, and FR_0.49_) due to time constraints. Flowering **(C)** and fruit ripening **(D)** rates were determined as the number of trusses per week with all flowers reaching anthesis or all fruits reaching ripe stage, respectively. Each data point represents the average of two experimental units ± SEM, where the value per experimental unit is the average of six plants **(A, C, D)** or 15 fruits **(B)**. FR, far-red light.

The fraction of dry matter partitioned to the fruits and potential fruit weight both showed a significant linear relationship with the FR fraction in supplementary lighting, despite the latter relationship being weaker (**Figures 2A,B**). In contrast, the total fruit number per plant did not show any significant trend across the FR gradient. Both flowering rate and fruit ripening rate were significantly affected by the FR fraction, with flowering rate showing a quadratic relationship and fruit ripening rate a linear relationship (**Figures 2C,D**). Despite these effects, both parameters increased by only +4% on average between the lowest and highest FR treatments.

### 3.3 Plant dry weight, photosynthesis, and canopy architecture

**Figure 3:**
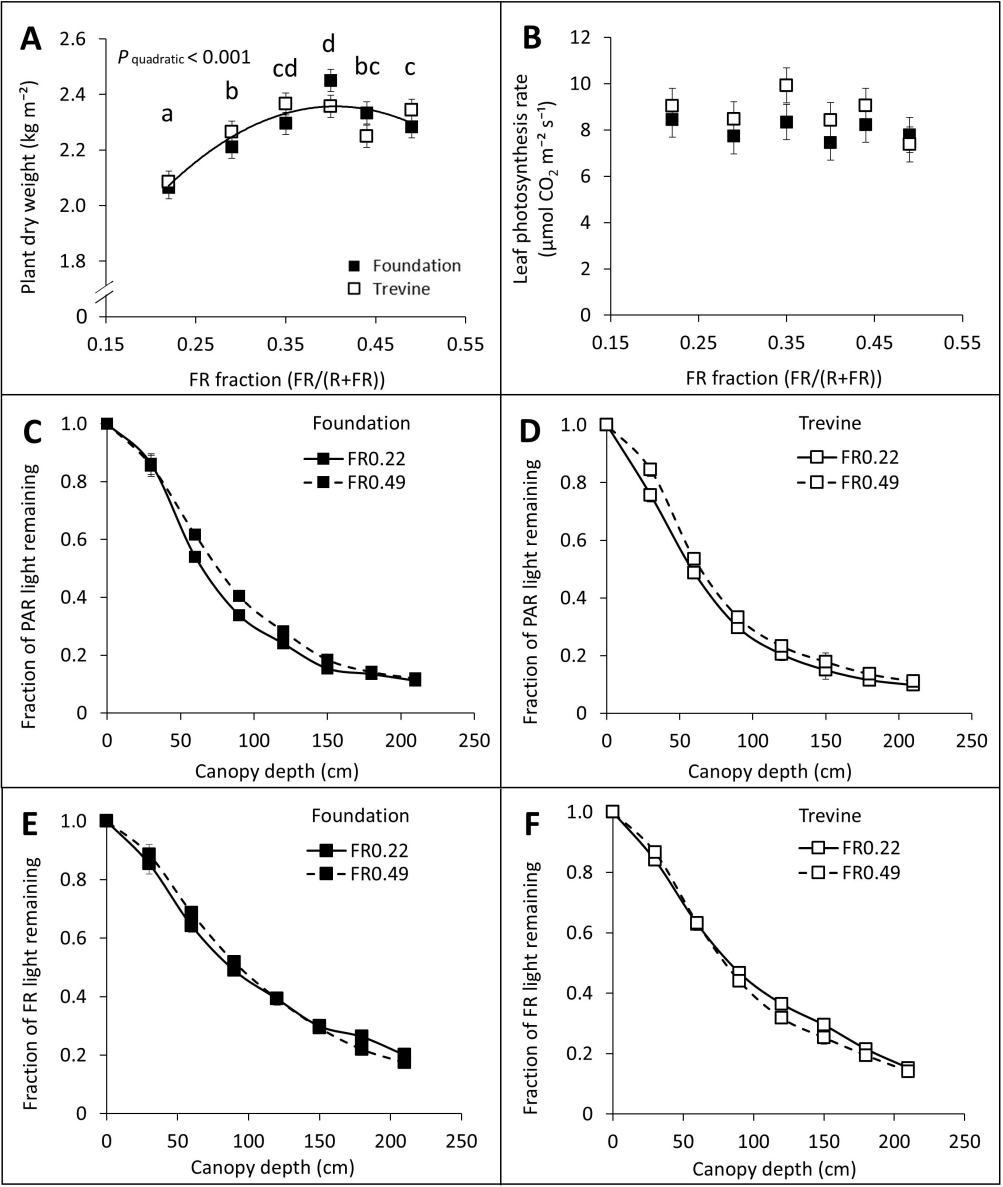
Effects of FR fraction in supplementary light on plant dry weight after 20 weeks of cultivation, 140–143 DAT **(A)**, leaf photosynthesis rate measured between 128 and 134 DAT **(B)**, fraction of PAR **(C, D)**, and FR **(E, F)** light remaining at different canopy depths for cv. Foundation and cv. Trevine. A trendline is depicted to show a significant quadratic relationship between plant dry weight and FR fraction (p < 0.1, averaged over both cultivars), and letters denote significant differences between treatments, as determined by Fisher’s protected LSD test. Each data point represents the average of two experimental units ± SEM, where the value per experimental unit is the average of five **(B)** or six **(A)** plants or the average of two experimental units **(C-F)**. FR, far-red light; DAT, days after transplant.

Similarly to fruit yield, plant dry weight increased with increasing FR fraction in supplementary light up to FR_0.40_ (+16% on average between the two cultivars, compared to FR_0.22_, **Figure 3A**). The leaf photosynthesis rate did not show any significant relationship with the FR fraction in supplementary light (**Figure 3B**). The FR treatments appeared to impact the vertical PAR light distribution within the canopy. In the top 150 cm of the canopy, a higher fraction of PAR light was retained under FR_0.49_ compared to FR_0.22_ for both cultivars (**Figures 3C,D**), resulting in faster light extinction in canopies developed under FR_0.22_. Consistent with this, the PAR light extinction coefficient exhibited a negative trend with increasing FR fraction (−7% to −8%, FR_0.49_ vs. FR_0.22_), although this trend was not statistically significant (**Supplementary Figure S7**). Notably, the vertical distribution of FR light within the canopy differed slightly from that of PAR (**Figures 3E,F**). The FR treatments had a limited impact on the fraction of FR light retained in the upper canopy, mostly present in cv. Foundation, while a higher proportion of FR light was retained in the lower canopy for the lowest FR treatment (FR_0.22_). This resulted in a small difference in FR light interception between treatments, with FR_0.49_ intercepting 5% to 8% more FR light than FR_0.22_ for cv. Trevine and cv. Foundation, respectively. Overall, almost 90% of incident PAR light was intercepted by the tomato canopy, whereas only 81% of incident FR light was intercepted. Leaf area index and average leaf area displayed a significant interaction between FR fraction and cultivar, with cv. Trevine showing a decrease across the FR gradient, whereas cv. Foundation showed a slight increase (**Supplementary Figure S8**). Interestingly, specific leaf area decreased linearly across the FR gradient for both cultivars. Stem length at the end of the experiment showed a significant linear increase with the FR fraction. However, the overall increase in stem length between FR_0.22_ and FR_0.49_ was relatively modest, only 28 cm on average (+5%) after 20 weeks of cultivation.

### 3.4 Yield component analysis

**Figure 4:**
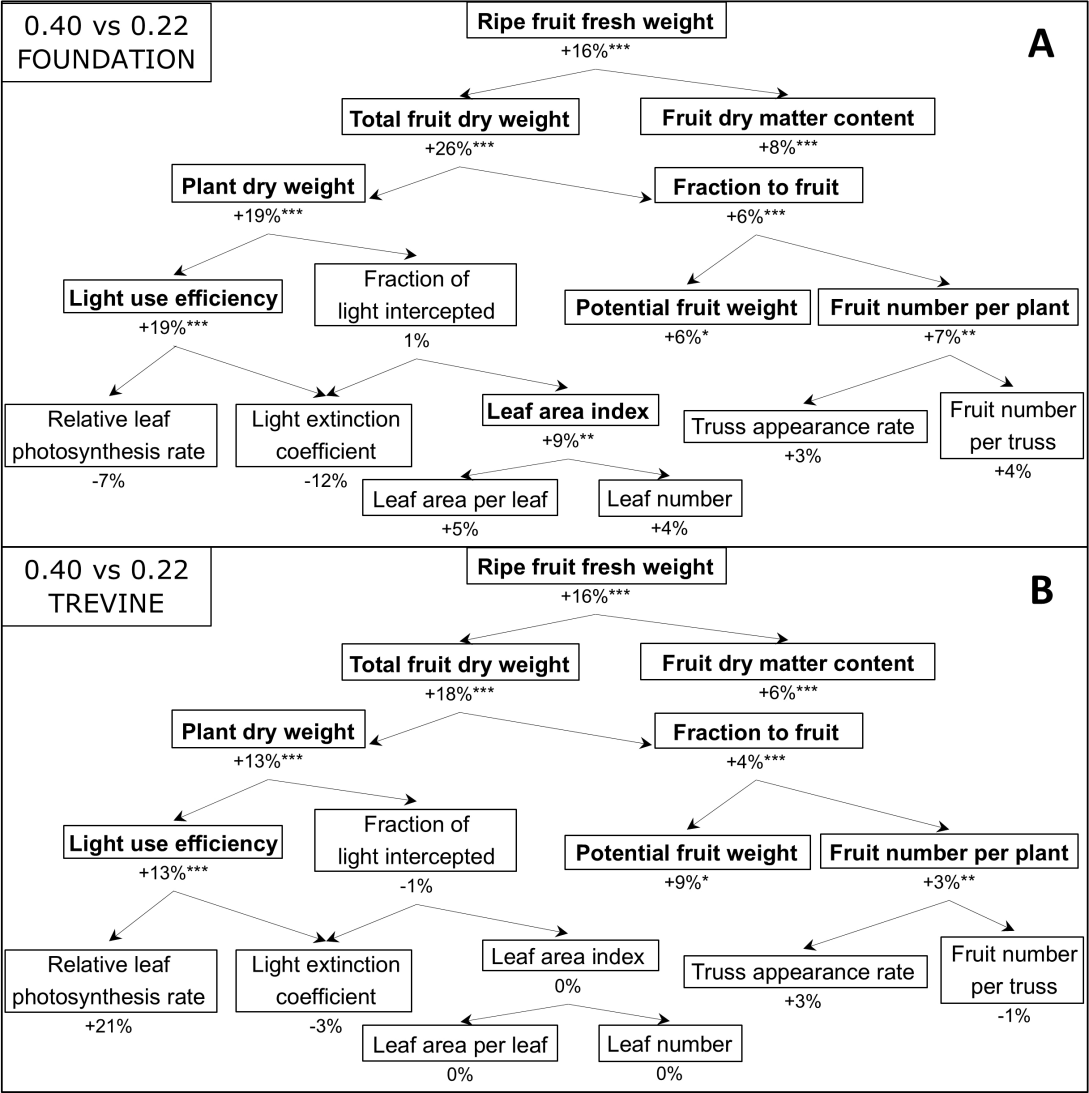
Yield component analysis representing the effects of FR_0.40_, compared to FR_0.22_, for cv. Foundation **(A)** and cv. Trevine **(B)**. The effect of additional FR is represented through the percentage difference between FR_0.22_ and FR_0.40._ Relative leaf photosynthesis rate was obtained by dividing the leaf photosynthetic rate by the incident PPFD, and it was measured between 128 and 134 DAT. All other data derive from the final destructive harvest (140 DAT) or represent averages and cumulative sums across the entire experimental period. Asterisks indicate a significant effect of FR_0.40_ as determined by Fisher’s protected LSD test (*p < 0.1; **p < 0.05; ***p < 0.01). FR, far-red light; PPFD, photosynthetic photon flux density; DAT, days after transplant.

We assessed how the FR gradient affected the components contributing to tomato fruit yield through a yield component analysis, comparing the FR fraction treatments resulting in the highest and lowest fruit yields (FR_0.40_ versus FR_0.22_, **Figure 4**). The addition of 45 μmol m^−2^ s^−1^ of supplementary FR promoted fruit yield by +16% and fruit dry matter content by +6% to 8% in both cultivars. The FR effect on total fruit dry weight (ripe + unripe fruits) was higher than on fruit yield for both cultivars, with Foundation showing the largest increase (+26% FR_0.40_ versus FR_0.22_, **Figure 4A**). This was connected to an increase in plant dry weight (+19% for cv. Foundation and +13% for cv. Trevine) and a greater fraction of dry matter partitioned to the fruits. The higher fraction of dry matter partitioned to the fruits was influenced by a significant increase in potential fruit weight, defined as the fruit weight under unlimited assimilate supply and used to quantify fruit sink strength (Marcelis, 1996), and by a higher fruit number per plant (ripe + unripe fruits). Interestingly, the increase in plant dry weight was not associated with any increase in the fraction of PAR light intercepted by the plant canopy, despite the positive increase in leaf area index for cv. Foundation (**Figure 4A**). A significant increase in plant light use efficiency was associated with a decrease in light extinction coefficient in both cultivars, resulting in a higher percentage of PAR light penetrating through the canopy (**Figures 3C,D**). The effect of FR_0.40_ on the relative leaf photosynthesis rate (leaf photosynthesis rate/incident PPFD inside the transparent measurement chamber) was positive for cv. Trevine and negative for cv. Foundation, although neither was statistically significant. When comparing the treatments with the lowest and highest FR fractions (FR_0.49_ vs. FR_0.22_, **Supplementary Figure S8**), the yield component analysis remains largely unchanged, although the magnitude of the treatment effects decreases for almost all parameters, with the notable exception of fruit dry matter content and the fraction of dry matter partitioned to the fruits, which tend to increase.

### 3.5 Fruit quality at harvest

**Figure 5:**
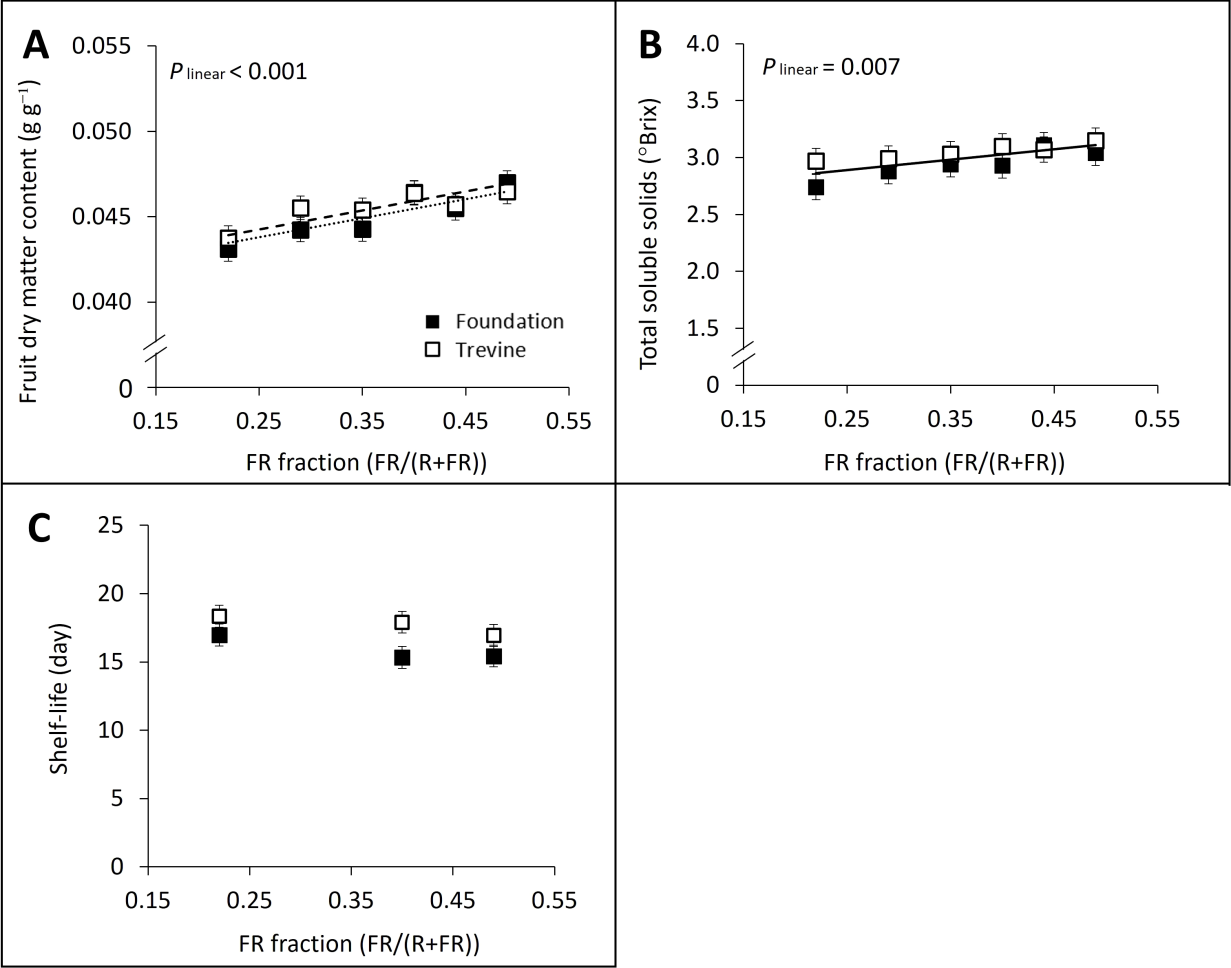
Effects of FR fraction in supplementary light fruit dry matter content **(A)**, total soluble solids content **(B)**, and shelf-life **(C)** for cv. Foundation and cv. Trevine. Trendlines are depicted to show a significant linear relationship with the FR fraction (p < 0.1; dashed line for cv. Trevine, dotted line for cv. Foundation, and solid line when there is no significant cultivar effect). Each data point represents the average of two experimental units ± SEM, where the value per experimental unit is the average of 18 **(A, B)** and 27 **(C)** fruits. FR, far-red light.

The fruit dry matter content and total soluble solids content (°Brix) of ripe fruits at harvest showed a significant linear relationship with the FR fraction in supplementary light (**Figures 5A,B**) and a significant positive correlation with each other (R^2^ = 0.55, p = 0.006, **Supplementary Figure S9**). The pH of harvested fruits followed a similar positive linear relationship with the FR fraction in supplementary light (p_lin_ = 0.078), although the effects were small (up to +2% for both cultivars). In contrast to the other fruit quality variables, soluble sugar concentration did not show a significant relationship with the FR fraction but showed a quadratic interaction between the two factors of the ANOVA, FR fraction × cultivar. Cv. Foundation recorded its highest sugar concentration at FR_0.49_, with only a 2% increase compared to the low FR treatment (FR_0.22_), while cv. Trevine reached its peak sugar concentration at FR_0.40_, showing a 12% increase over FR_0.22_. Similar trends were observed when glucose and fructose concentrations were analysed separately. Sucrose concentration was too low in ripe fruits to display significant differences among the treatments. Shelf-life was defined as the length of days of high marketability for the tomato fruits. Shelf-life was not significantly affected by the FR fraction, although it was 1 day shorter at FR_0.49_ compared to FR_0.22_ (**Figure 5C**).

## 4 Discussion

### 4.1 Fruit yield response to FR supplementation shows a non-linear trend at higher FR fractions

This research aimed to quantify the relationship between the FR fraction in supplementary lighting and tomato fruit yield. A high-wire tomato crop was grown under a gradient of FR fractions, from 0.22 to 0.49, realised by increasing supplementary FR while keeping the supplementary PAR constant (250 µmol m^−2^ s^−1^). From FR_0.22_ to FR_0.40_, fruit yield increased by 16% (**Figure 4**), but exceeding FR_0.40_ resulted in no further benefit, decreasing fruit yield by 2% and 9% at FR_0.49_ for cv. Trevine and cv. Foundation, respectively. These findings are consistent with previous studies reporting increases in fruit yield at low levels of FR supplementation (Ji et al., 2019; Kim et al., 2019; Vincenzi et al., 2024) and provide novel insight into the upper threshold of FR supplementation beyond which further yield improvement is not observed. We hypothesised that fruit yield would only show a linear response at lower levels of FR supplementation, reaching an optimum or saturation point at higher FR fractions. Our results showed that fruit yield response followed a linear relationship only up to FR fraction 0.40 (**Figure 1A**). However, they were not conclusive in determining whether the response followed a saturation or optimum curve across the whole FR gradient. In particular, fruit yield (ripe fruit fresh weight) response suggests an optimum around FR fraction 0.40, especially for cv. Foundation, while ripe fruit dry weight aligns more closely with a saturation response (**Figures 1B, C**). Interestingly, fruit dry matter content increased linearly with the FR fraction, indicating a consistent reduction in fruit water content across the full FR gradient (**Figure 5A**). Increases in fruit dry matter content under high FR could be linked to enhanced sugar and starch accumulation (Ji et al., 2020; Dorokhov et al., 2021), along with reduced water dilution (Fanwoua et al., 2019). Although the mechanism behind the FR effect on fruit dry matter content remains unclear, proposed explanations include changes in the balance of xylem and phloem water import, altered phloem sap concentration, or increased fruit transpiration (Fanwoua et al., 2019). These dynamics may explain the different trends observed between fruit fresh and dry weight at high FR fractions, where dry weight remained stable but fruit water content decreased, negatively affecting fresh weight.

In our experiment, the PAR (400–700 nm) daily light integral (DLI) of supplementary lighting was kept constant across the treatments, while the FR fraction [FR/(R + FR)] was varied among treatments by changing supplementary FR (700–800 nm). Solar radiation also entered into the greenhouse, but it was only on average 15% of the total PAR DLI (solar + supplementary lighting) and 13% to 30% of the total FR DLI for treatment FR_0.49_ and FR_0.22_, respectively. The impact of solar radiation was the highest during the first 4 weeks after transplant when the supplementary lighting had not yet reached the full photoperiod of 16 h (**Supplementary Figure S3**). Once supplementary lighting reached the full photoperiod, solar radiation accounted for only 7.5% of total PAR DLI and 6% to 18% of total FR DLI, on average. Solar radiation could potentially influence our light treatments by altering the light intensity and spectral quality perceived by the plants. Specifically, solar radiation increases the total PAR DLI, and several studies have reported that FR effects on photosynthesis and plant morphology can depend on the background light intensity (Wassenaar et al., 2022; Kusuma and Bugbee, 2023; Lazzarin et al., 2024; Shomali et al., 2024). Moreover, solar radiation has a relatively high FR fraction (~0.46), which can affect the overall FR fraction (solar + supplementary lighting) perceived by the plants, particularly for the light treatments with the lowest FR fractions (FR_0.22_ and FR_0.29_). However, while the DLI from solar radiation was the highest in the first part of the growth period and then declined, the DLI from supplementary lighting followed the opposite trend. This resulted in a relatively stable total PAR DLI of approximately 15 mol m^−2^ day^−1^ throughout the experiment (**Supplementary Figure S3**). Finally, the effect of solar radiation on the overall FR fraction perceived by the plants was quantified (**Table 1**), and it was found that accounting for this contribution did not change the results of our statistical analysis (**Supplementary Figure S10**). Therefore, we concluded that the presence of solar radiation in our experimental setup did not significantly influence the effects of the light treatments.

To evaluate the performance of additional FR lighting under constant PFD conditions, we compared the radiation and electricity use efficiency (RUE and EUE, respectively) determined by increasing doses of FR with simulated RUE and EUE based on increasing doses of PAR (**Figures 1D, E**). We derived the simulated fruit yield under additional PAR using the relationship between tomato fruit fresh weight and PAR light integral (average 0.85% increase in fruit fresh weight for each 1% increase in PPFD; Marcelis et al., 2006). The results suggest that up to FR_0.40_, additional FR can result in a comparable increase in fruit yield as additional PAR while consuming less electricity for supplementary lighting. However, these findings should be interpreted in view of the spectral characteristics of the lighting fixtures we used. The electrical consumption of an LED lighting fixture is strongly influenced by its spectrum, with far-red and red LEDs having the highest potential efficacies (kWh of electricity consumed per µmol of photons emitted; Kusuma et al., 2020). In this study, PAR supplementary lighting was supplied by white-spectrum fixtures with relatively low red content (45%), which facilitated the implementation of the FR fractions. Commercial tomato cultivation typically relies on LED lighting with a spectrum containing 90% of red photons or more, which would result in EUE values up to ~50% higher than those obtained here (considering red + blue fixtures with a current maximum efficacy of 3.7 µmol J^−1^, instead of the white-spectrum fixtures used in this study, which had an efficacy of 2.5 µmol J^−1^). Additionally, it is important to note that in this study, RUE and EUE were lumped parameters integrating FR effects on leaf photosynthesis rate, plant architecture, and dry matter partitioned to the fruits. Previous research on the same tomato cultivars reported that the RUE of supplementary lighting was not significantly affected by increasing the duration of FR application, with an FR fraction between 0.10 and 0.28 and a supplementary PPFD of approximately 230 µmol m^−2^ s^−1^ (Vincenzi et al., 2024). In this study, we extended the tested range of FR fractions up to 0.49 and observed a decrease in RUE and EUE only at the highest levels (0.44 and 0.49). At these levels, further FR supplementation appears to be ineffective in improving fruit yield. This result aligns with earlier studies, which reported no yield benefit from supplementary FR (8 to 36 µmol m^−2^ s^−1^ added to a supplementary PPFD of 144 to 170 µmol m^−2^ s^−1^) under conditions where solar radiation contributed a large portion of the total PFD (Hao et al., 2016; Dzakovich et al., 2017; Palmitessa et al., 2020).

### 4.2 Impact of FR supplementation on fruit yield components varies with FR fraction

The increase in fruit production was primarily associated with higher plant dry weight, which showed a similar response to FR fraction as fruit yield (+13% to 19% at FR_0.40_, **Figure 4**). Although the fraction of dry weight partitioned to the fruits linearly increased with additional FR, treatment differences were rather small (up to +5% to 6% when comparing FR_0.49_ versus FR_0.22_). This finding contrasts with previous yield component studies, where FR effects on fruit yield were largely attributed to increased dry matter partitioned to the fruits (Ji et al., 2019, 2020) or equally attributed to increased plant dry weight and increased dry matter partitioned to the fruits (Vincenzi et al., 2024). The main reason for this discrepancy may lie in the FR fraction of the light treatments tested. The lowest FR treatment in our experiment (FR_0.22_, **Table 1**) has an FR fraction similar to the highest FR fraction included in previous studies (up to FR fraction 0.26–0.28, Ji et al., 2019; Vincenzi et al., 2024). Therefore, the relative contribution of each of these two components to the FR-mediated increase in fruit yield may depend on the FR fraction in supplementary light.

### 4.3 FR effect on plant dry weight does not clearly correlate with changes in leaf photosynthesis or canopy architecture

The increase in plant dry weight was associated with an increase in plant light use efficiency (plant dry weight per unit of solar and supplemental PPFD), while the fraction of PAR light intercepted was not affected by the FR fraction (**Figures 4** and **S8**). A tomato canopy with an Leaf Area Index (LAI) of 3 intercepts approximately 90% of incident light (Heuvelink, 1996). We determined leaf area index at 5, 16, and 20 weeks after transplant and found that LAI exceeded 3 for both cultivars and all treatments from the earliest measurement onwards, never dropping below this threshold at any subsequent time point. Thus, the moderate changes in leaf area recorded in this study (<10%) did not affect PAR light interception, which already approached 90% on average.

The higher light use efficiency when plants were grown under a higher fraction of FR represented the combined effects of FR on leaf photosynthesis rate and light distribution within the canopy, although neither parameter was statistically significant in our measurements (**Figure 4**). Far-red (particularly between 700 and 750 nm) can synergise with shorter wavelengths to enhance the efficiency of photochemistry by balancing the excitation of the two photosystems, resulting in a higher leaf photosynthesis rate and photosystem II operating efficiency (Emerson and Rabinowitch, 1960; Hogewoning et al., 2012; Zhen and van Iersel, 2017; Lazzarin et al., 2024). This short-term enhancement effect of FR on photosynthesis is expected to decrease at higher FR doses (above 0.4 FR/PPFD, or in this experiment above FR fraction 0.47), as sufficient FR is present to balance photochemistry (Zhen and Bugbee, 2020). Concurrently, long-term acclimation to FR-enriched environments can reduce chlorophyll content, leaf absorbance, and maximum photosynthesis at saturating light intensity (Ji et al., 2019; Dorokhov et al., 2021; Wassenaar et al., 2022; Vincenzi et al., 2024). In this study, no significant relationship was found between the FR fraction in supplementary light and leaf photosynthesis (**Figure 3B**). The lack of a measurable FR effect in our photosynthesis data may be due to i) an overlap of both short-term and long-term acclimations to increasing FR fractions, which may counterbalance each other, and ii) a decrease in the short-term enhancement of photosynthesis in the treatments with the highest FR fractions, as they were close to and above the threshold of 0.4 FR/PPFD (Zhen and Bugbee, 2020). Finally, PAR light extinction coefficients for both cultivars decreased with increasing FR fraction in supplementary light, although not significantly (**Supplementary Figure S7**). This reduction may be due to an FR effect on canopy architecture (such as elongated stem internode or leaf petiole) or an FR-mediated decrease in leaf absorbance due to lower chlorophyll content. A lower light extinction coefficient indicates a more uniform vertical light distribution, with less light absorbed by the leaves high in the canopy, that operate closer to light saturation. This can increase canopy photosynthesis by making more light available to leaves lower in the canopy operating more in the linear part of the photosynthesis curve (Li et al., 2014; Schipper et al., 2023).

### 4.4 Fruit quality at harvest and fruit shelf-life was not substantially affected by the FR treatments

We assessed fruit quality at harvest and shelf-life to determine whether the FR effect on fruit yield would alter fruit quality. Fruit dry matter content increased linearly with the FR fraction in supplementary lighting up to +9% for cv. Foundation at FR_0.49_. This finding aligns with previous studies reporting that growing tomato fruits in FR-enriched environments increases their dry matter content (Fanwoua et al., 2019; Kim et al., 2019, 2020; Dorokhov et al., 2021) and that this increase follows a linear relationship with the dose of FR (Vincenzi et al., 2024). Total soluble solids (°Brix) content displayed a positive and significant correlation with fruit dry matter content (**Supplementary Figure S9**). Both variables are closely associated with the sweetness component of tomato taste, as soluble sugars, primarily glucose and fructose, account for approximately 50% of the dry weight of ripe tomatoes (Ho, 1996; Beckles, 2012). However, the maximum increase observed in this study was only 0.37°Brix, making it unlikely to result in noticeably sweeter tomatoes. FR fraction in supplementary light during cultivation did not significantly affect tomato shelf-life (**Figure 5C**). Shelf-life was evaluated qualitatively, considering changes in colour, shape, and firmness over time for fruit stored in darkness under high relative humidity (>80%), at 20°C. Previous research on tomatoes grown with intercanopy FR lighting also reported no significant differences in colour, firmness, or weight loss after 7 days of storage at 13°C and high relative humidity (Appolloni et al., 2023). Overall, the effects of FR on fruit quality in this experiment were small, when present, and are unlikely to impact the consumer perception of tomato taste or its shelf-life.

## 5 Conclusions

Tomato fruit yield increased linearly with the addition of FR to supplementary lighting, but only at lower FR fractions (below 0.40). Within this range, FR addition leads to similar yield increases as calculated for adding the same moles of PAR. However, at higher FR fractions, further increases in FR resulted in diminishing yield gains or even yield reductions, which in turn led to decreased radiation and electricity use efficiency. Adding FR did not significantly impact fruit quality at harvest nor shelf-life in a way likely to influence consumer preference, even at high FR fractions. Finally, the increase in fruit yield across most of the FR fraction gradient (0.22 to 0.40) was primarily linked to increased plant dry weight, while the effect on dry matter partitioned to the fruits was positive but smaller.

## Data Availability

The raw data supporting the conclusions of this article will be made available by the authors, without undue reservation.
