# Correction: Dose-response of tomato fruit yield to far-red fraction in supplementary lighting

**DOI:** 10.3389/fpls.2025.1701163

**Published:** 2025-10-10

**Authors:** Elena Vincenzi, Aron Moehn, Emmanouil Katsadas, Sana Karbor, Esther de Beer, Frank Millenaar, Leo F M Marcelis, Ep Heuvelink

**Affiliations:** ^1^ Horticulture and Product Physiology, Department of Plant Science, Wageningen University and Research, Wageningen, Netherlands; ^2^ Signify Netherlands B.V., Eindhoven, Netherlands; ^3^ BASF–Nunhems, Nunhem, Netherlands

**Keywords:** tomato, far-red light, radiation use efficiency, electricity use efficiency, fruit quality, vertical light distribution, photosynthesis, yield component analysis

In the published article, there was an error in **Figure 3** as published. **Figure 3B** depicted the effects of FR fraction in supplementary light on the fraction of dry matter partitioned to fruits instead of leaf photosynthesis rate. The corrected **Figure 3** and its caption appear below.

**Figure 3:**
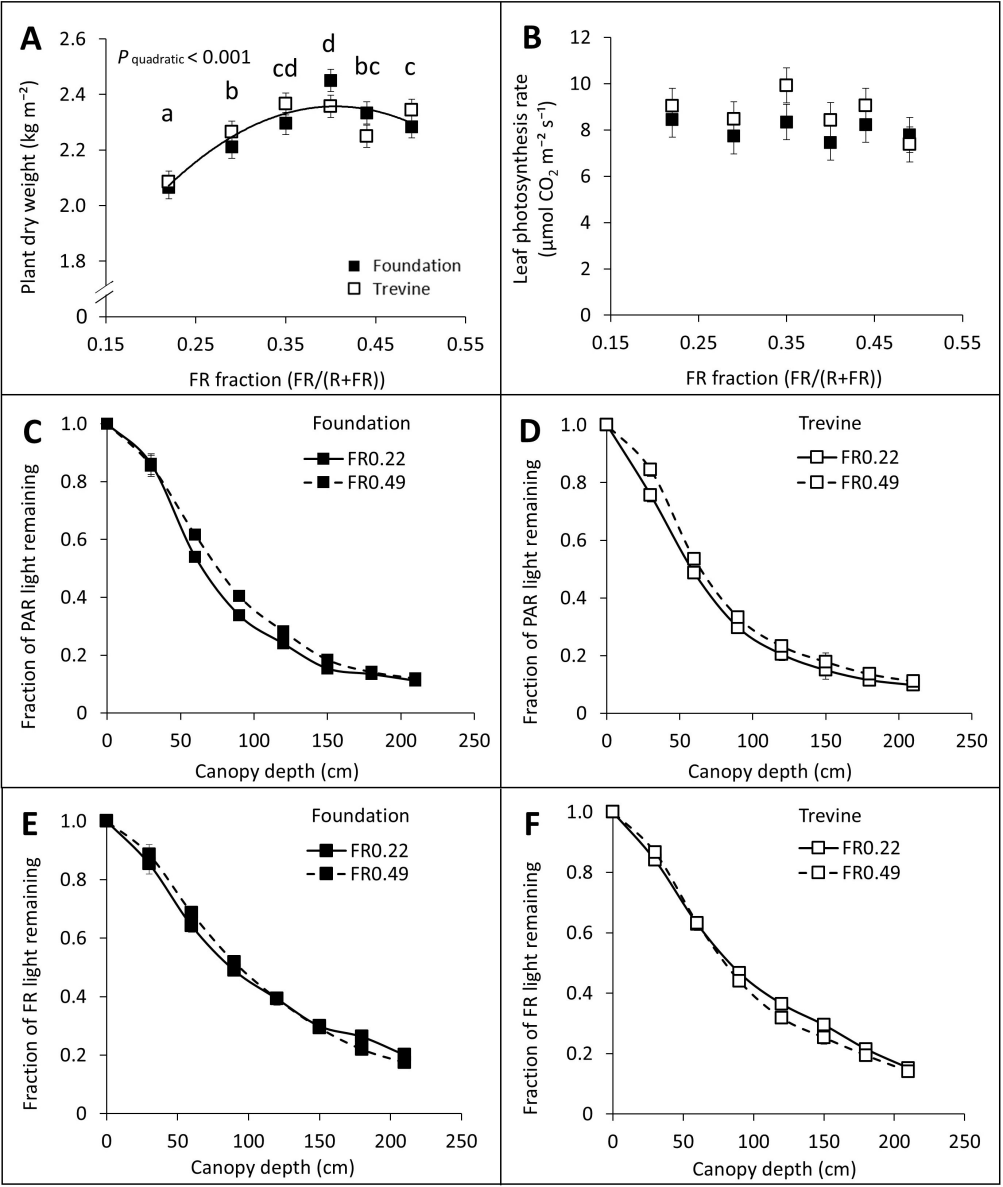
Effects of FR fraction in supplementary light on plant dry weight after 20 weeks of cultivation, 140–143 DAT **(A)**, leaf photosynthesis rate measured between 128 and 134 DAT **(B)**, fraction of PAR **(C, D)**, and FR **(E, F)** light remaining at different canopy depths for cv. Foundation and cv. Trevine. A trendline is depicted to show a significant quadratic relationship between plant dry weight and FR fraction (p < 0.1, averaged over both cultivars), and letters denote significant differences between treatments, as determined by Fisher’s protected LSD test. Each data point represents the average of two experimental units ± SEM, where the value per experimental unit is the average of five **(B)** or six **(A)** plants or the average of two experimental units **(C-F)**. FR, far-red light; DAT, days after transplant.

The original article has been updated.

