# Supplementary material for: Dose-response of tomato fruit yield to far-red fraction in supplementary lighting
Source: Front Plant Sci. 2025 Jul 10;16:1618171. doi: 10.3389/fpls.2025.1618171 (PMC12287650; doi:10.3389/fpls.2025.1618171)
Supplement: Supplementary file 1 [file DataSheet1.docx]

# ***Supplementary Material***

# **Supplementary Figures**

**
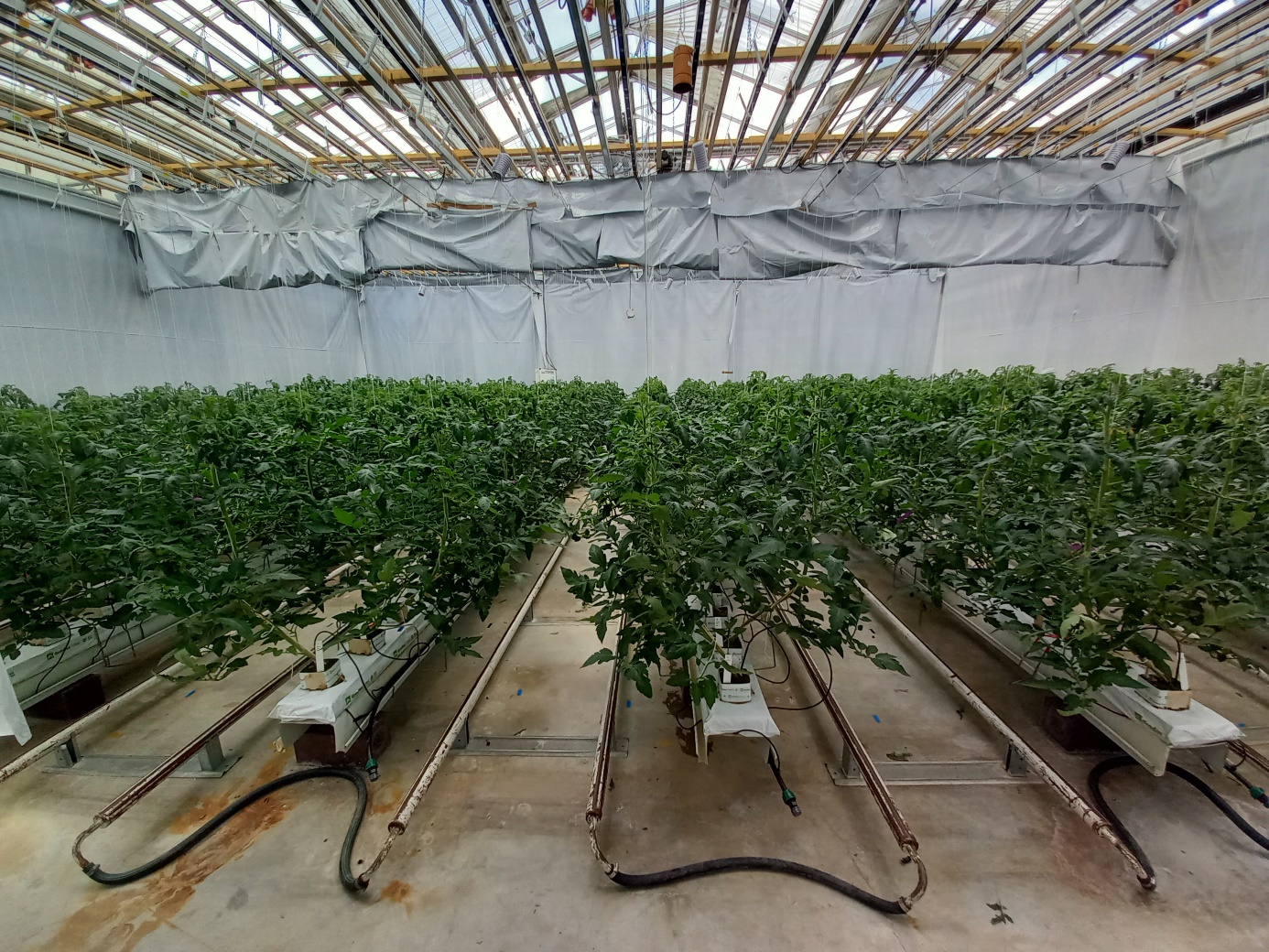
**

**Supplementary Figure S1.** The figure shows one of the two greenhouse compartments used in this experiment. In the visible (front) half of the compartment, the far-red fraction increases along a gradient from left to right. At the top, the wooden framework necessary to support the far-red and white light modules, while maintaining uniform shading across the entire compartment. Below, a 1-meter-long plastic curtain separates the light environments of the front and back halves of the compartment.

**
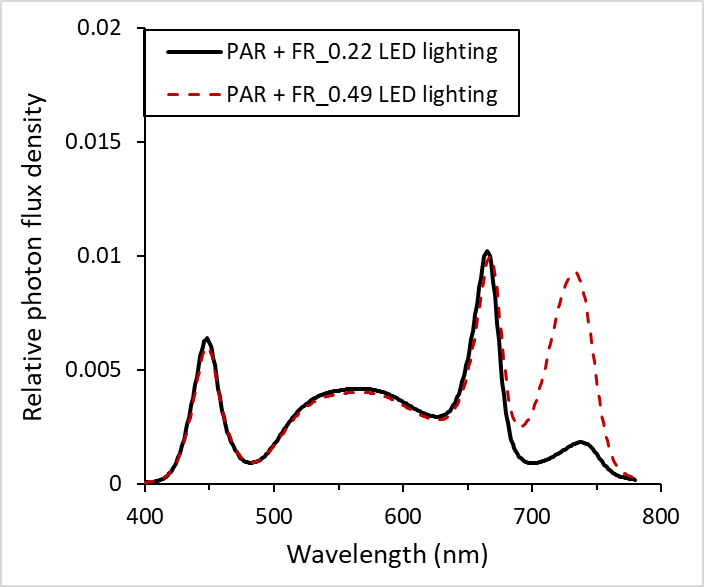
**

**Supplementary Figure S2.** The spectral composition of the supplementary photosynthetically active radiation and far-red (PAR + FR) LED lighting as measured in the two extreme treatments of the FR gradient (FR_0.22_ and FR_0.49_). Measurements were carried out with a spectrometer at 2 m above the ground, before transplant.

**
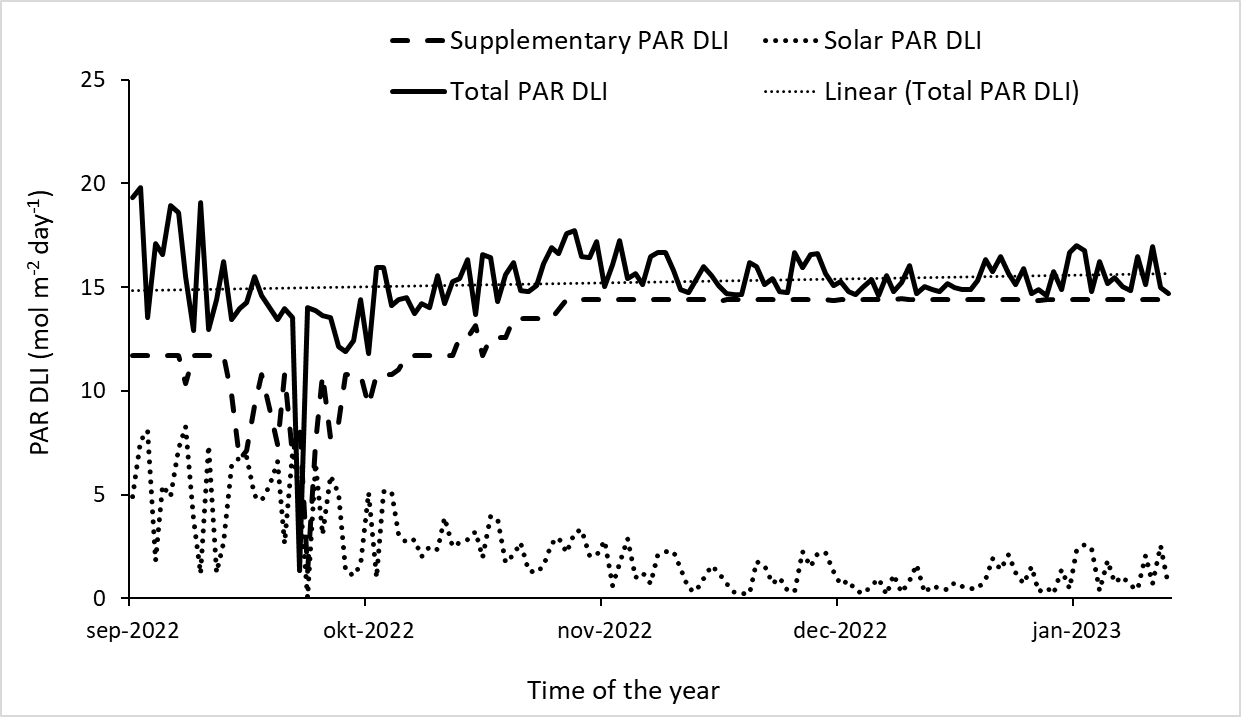
**

**Supplementary Figure S3.** Photosynthetically active radiation daily light integral (PAR DLI) as determined by solar and supplementary light during the course of the greenhouse experiment. The contribution of supplementary lighting was determined from the average light intensity supplied from the LED modules, measured at 2 m above the ground, and the daily hours of lamp activity. The contribution of solar light was determined from the solar light measurements taken with a solarimeter on the roof of the greenhouse and the greenhouse transmissivity coefficient. Total light is calculated as the sum of the contributions by solar light and supplementary lighting. A trendline is displayed for total PAR DLI data. On October 8^th^, 2022, all lights were turned off for maintenance, determining a temporary dip in supplementary PAR DLI.


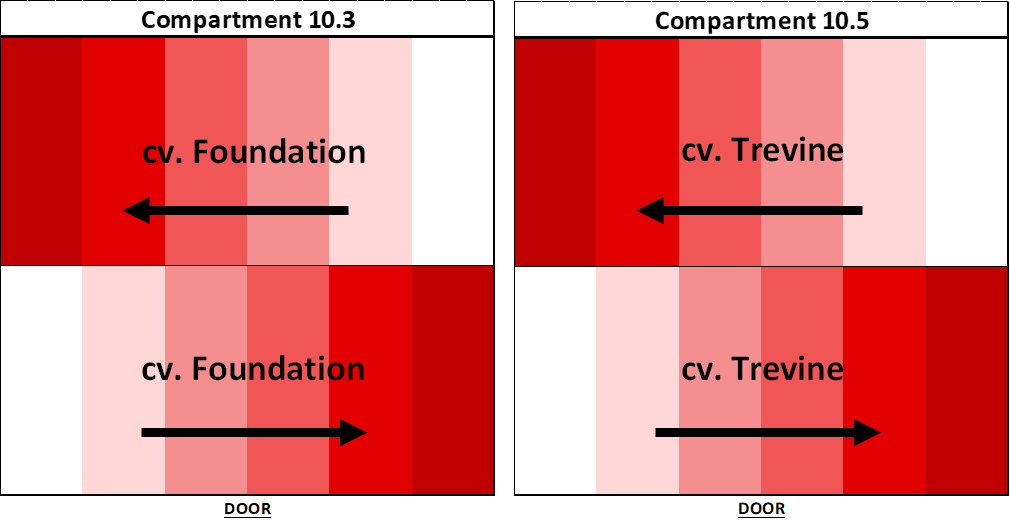


**
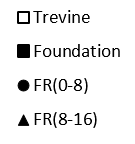
Supplementary Figure S4.** Experimental setup in two greenhouse compartments 10.3 and 10.5. The increase in FR light intensity is represented by darker shades of red and by the direction of the arrow in each replication of the gradient. All supplementary lighting was provided by overhead LED modules mounted on a wooden frame.

*
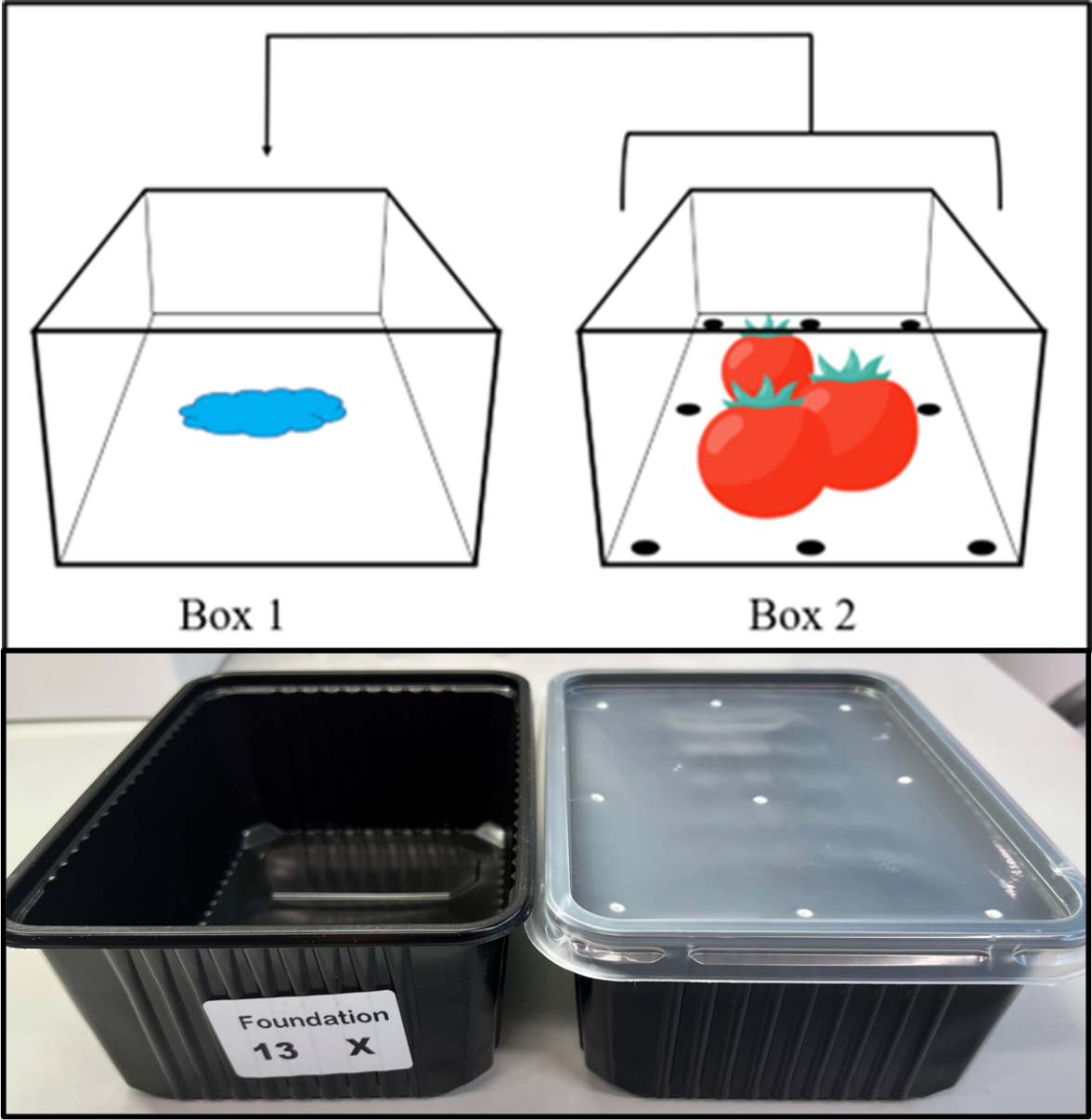
*

**Supplementary Figure S5.** Shelf-life setup. During the shelf-life measurement, each biological replicate was stored in a setup designed to maintain high relative humidity. The setup consisted of two disposable plastic containers (18 x 16 x 6.5 cm), one placed inside the other. The outer container (Box 1) held a small amount of water, which was checked every time fruit quality was assessed and replaced as needed. The inner container (Box 2) had eight holes (3–5 mm in diameter) at the bottom, allowing water from the outer container to evaporate and maintain high humidity for the tomatoes without direct contact. A plastic lid with 9 small holes (1 mm in diameter) was placed on top of the inner container to allow for air exchange. Each setup was kept in a climate cabinet (Technisch Buro I.K.S. LS700R, Leerdam, the Netherlands) at 20°C. The treatments were anonymized and randomized to prevent bias during scoring.

**
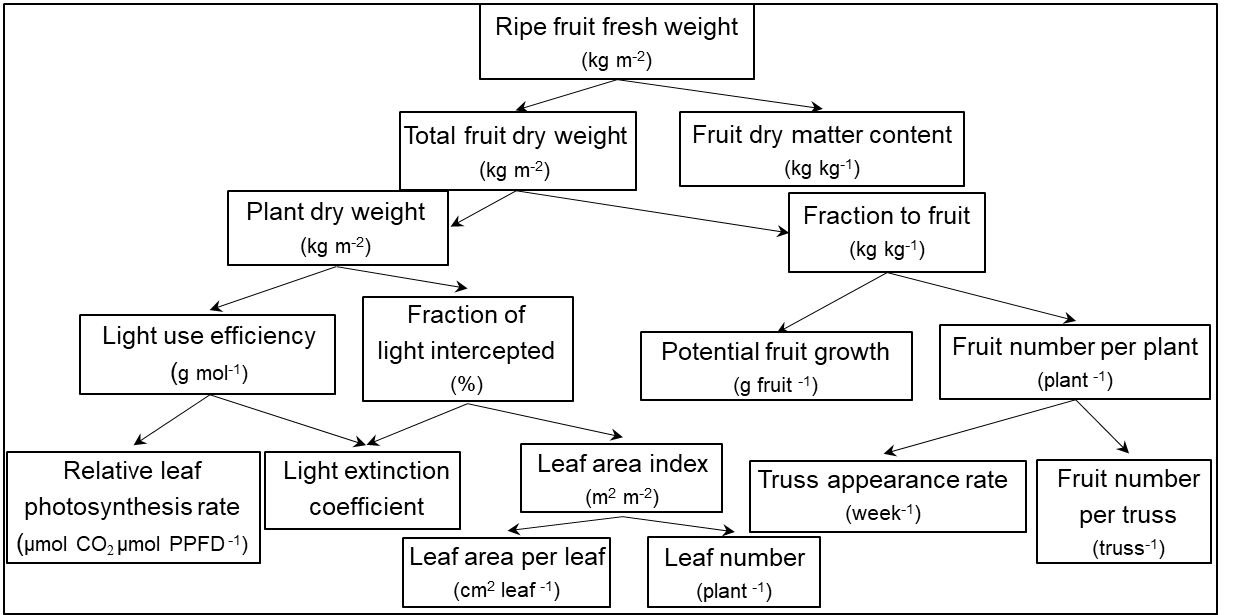
**

**Supplementary Figure S6**. Yield component analysis scheme, adapted from Vincenzi et al. (2024) . The scheme illustrates the physiological components that determine ripe fruit fresh weight. Light use efficiency was calculated as the ratio of plant dry weight at the end of the experiment and intercepted PPFD. Relative leaf photosynthesis rate was calculated by dividing the instantaneous photosynthesis rate by the incident PPFD. Potential fruit growth represents the average fruit dry weight obtained under non-limiting assimilate supply (one fruit per truss).

***
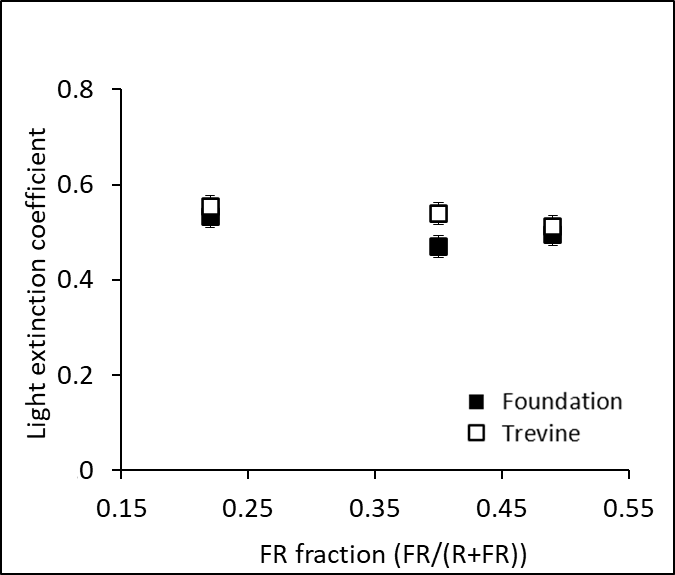
***

**Supplementary Figure S7.** Effect of FR fraction in supplementary light on light extinction coefficient for cv. Foundation and Trevine. Each data point represents the average of two experimental units ± SEM, where the value per experimental unit is the average of two measurement areas.
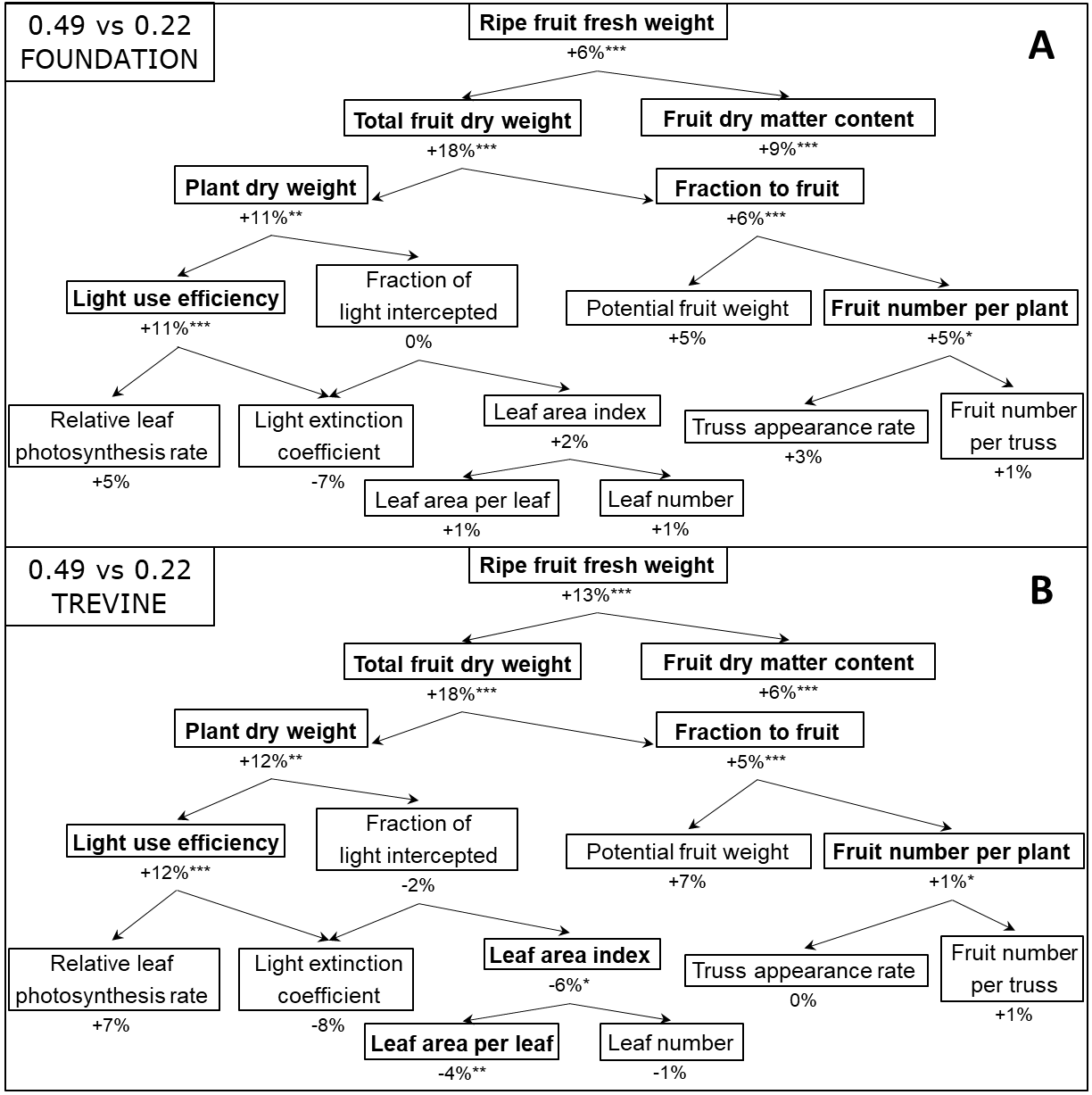


**Supplementary Figure S8.** Yield component analysis representing the effects of FR_0.49_, compared to FR_0.22_, for cv. Foundation (A) and Trevine (B).

The effect of additional FR was represented through the percentage difference between FR_0.22_ and FR_0.49._ The leaf photosynthesis rate was obtained by dividing the instantaneous photosynthetic rate by the incident PPFD, and it was measured between 128 and 134 DAT. All other data derive from the final destructive harvest (140 DAT) or represent averages and cumulative sums across the entire experimental period. Asterisks indicate a significant effect of FR_0.49_ as determined by Fisher’s Protected LSD test (* p <; ** p < 0.05; *** p < 0.01).


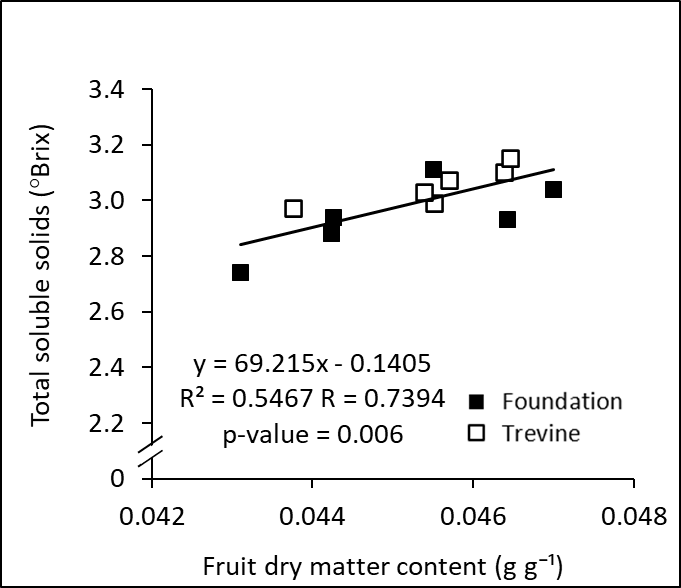


**Supplementary Figure S9**. Correlation between fruit dry matter content and total soluble solids content (°Brix) for cv. Foundation and Trevine. Each data point represents the average of two experimental units ± SEM, where the value per experimental unit is the average of 18 fruits. The graph shows the linear regression line between all the data, the coefficient of determination (R^2^) and the Pearson correlation coefficient (R) with the corresponding p-value.

*
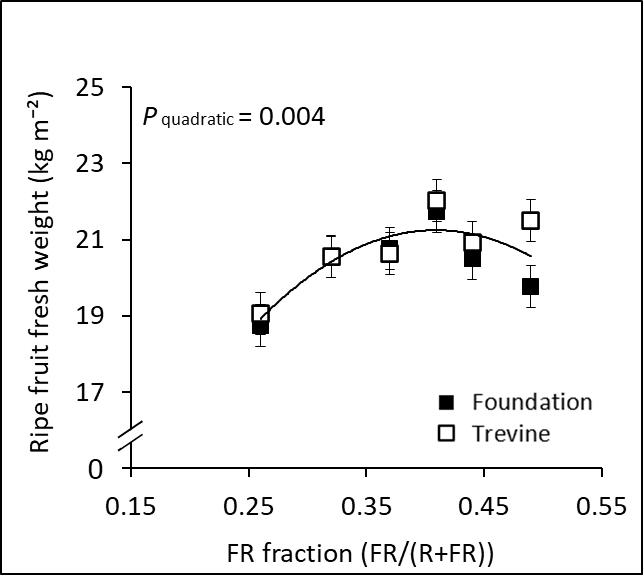
*

**Supplementary Figure S10**. Effects of FR fraction of total light (solar + supplementary light) on fruit yield (ripe fruit fresh weight). A trendline is depicted to show a significant quadratic relationship with the FR fraction (p < 0.1, averaged over both cultivars). Each data point represents the average of two experimental units ± SEM, where the value per experimental unit is the average of 6 plants. The data refers to cumulative values over a period of 10 weeks, from the first to the last fruit harvest, 63 to 139 DAT

# **Supplementary Tables**

**Supplementary Table S1.** Sunrise, sunset and activation times of white (W) and far-red (FR) LED modules per treatment throughout the experimental period.

| **Day After Transplant** | **Calendar Day** | **Sunrise^1^** | **Sunset^1^** | **W + FR on** | **W + FR off** |
| --- | --- | --- | --- | --- | --- |
| 0 | 13/9/22 | 07:11 | 20:00 | 06:50 | 19:45 |
| 7 | 20/9/22 | 07:22 | 19:43 | 06:40 | 19:35 |
| 14 | 27/9/22 | 07:34 | 19:27 | 05:20 | 18:15 |
| 21 | 4/10/22 | 07:46 | 19:10 | 06:05 | 18:00 |
| 28 | 11/10/22 | 07:58 | 18:54 | 05:50 | 17:45 |
| 35 | 18/10/22 | 08:10 | 18:39 | 05:35 | 17:30 |
| 42 | 25/10/22 | 08:23 | 18:24 | 04:25 | 17:20 |
| 49 | 1/11/22 | 07:36 | 17:11 | 02:10 | 16:05 |
| 56 | 8/11/22 | 07:48 | 16:58 | 01:00 | 15:55 |
| 63 | 15/11/22 | 08:01 | 16:47 | 00:20 | 16:15 |
| 70 | 22/11/22 | 08:13 | 16:39 | 00:10 | 16:05 |
| 77 | 28/11/22 | 08:24 | 16:32 | 00:05 | 16:00 |
| 84 | 7/12/22 | 08:35 | 16:27 | 00:00 | 15:55 |
| 91 | 14/12/22 | 08:43 | 16:26 | 00:00 | 15:55 |
| 98 | 21/12/22 | 08:48 | 16:28 | 00:00 | 15:55 |
| 105 | 28/12/22 | 08:50 | 16:33 | 00:05 | 16:00 |
| 112 | 4/01/23 | 08:49 | 16:40 | 00:10 | 16:05 |
| 119 | 11/01/23 | 08:46 | 16:50 | 00:20 | 16:15 |
| 126 | 18/01/23 | 08:40 | 17:01 | 00:30 | 16:25 |
| 133 | 25/01/23 | 08:32 | 17:13 | 00:45 | 16:40 |

^1^ Sunrise and sunset data for Amsterdam, the Netherlands (CET).

**Supplementary Table S2.** Scoring chart shelf-life measurement, adapted from Kader et al. (1973). The ‘Overall’ score is purely exemplary for the general appearance of the tomato and was not used throughout the experiment. The colour was scored with the aid of a tomato ripening colour chart (The Greenery, the Netherlands). Fruit firmness, colour and shape, were scored individually on a scale from 9 (highest quality) to 1 (lowest quality), with a score of 5 representing the threshold of reduced marketability. When the fruit quality index dropped below 5 for an entire replicate (three tomatoes in the same box), further observation of that replicate was terminated.

| Score | Overall | Firmness | Colour | Shape |
| --- | --- | --- | --- | --- |
| 9 | Excellent.  Typical for the freshly harvested tomato. | Firm tissue. No spots with reduced firmness. | Exceptionally appealing.  10 on the tomato ripeness scale. | Perfect, undamaged shape. |
| 8 | Very good.  Excellent with small imperfections.  Typical for the freshly harvested tomato. | Firm tissue. Occasional spots with slightly reduced firmness | Mostly red, yet a hint of orange or darker red is present.  10 with occasional imperfections at 11 on the tomato ripeness scale. | Very good shape. Small or hardly noticeable mechanical damage scars. |
| 7 | Good. | Mostly firm tissue.  Multiple spots with slightly reduced firmness. | Natural ripe red.  12 on the tomato ripeness scale. | Good shape. Occasional scars or imperfections. |
| 6 | Satisfactory.  A consumer would buy it in a retail shop, but not as the first choice | Mostly firm tissue. Frequent spots with reduced firmness. | 12 on the tomato ripeness scale, with occasional imperfections with a darker colour. | Acceptable shape. Scars and imperfections are always present, but not large. |
| 5 | Borderline.  A consumer would buy it in a retail shop, but only with a discount | Reduced firmness all over the fruit. | Significantly darker than 12 on the tomato ripeness scale. | Acceptable shape. Small cracks are present due to weight loss. |
| 4 | Unsatisfactory.  A consumer would not buy it in a retail shop. | Reduced firmness all over the fruit and some very soft spots. | Strong generalised discolouration across the fruit. | General loss of shape. Fruit has shrunken and/or cracked. Mould appears in mechanical damages/scars. |
| 3 | Poor. | Very soft fruit. | Complete discolouration. | Severe loss of shape. |
| 2 | Bad. | Unpleasantly soft fruit. | Towards repulsive colour. | Severe loss of shape. |
| 1 | Very bad. | Unpleasantly soft fruit. | Repulsive colour. | Shape has fully disintegrated. |

# **Supplementary Methodology**

**Supplementary method S1**. Soluble sugar content measurements

15 mg of freeze-dried fruit tissue is weighed into a 12 mL centrifuge tube, the precise weight is recorded. Ethanol extraction is performed by adding 5 mL of 80% ethanol to each sample. The tubes are vortexed before being incubated in a shaking water bath at 80°C for 20 minutes. After incubation, the samples are vortexed again.

Centrifugation follows at 8,500 rcf and 4°C for 5 minutes. 1 mL of the resulting supernatant is then transferred to 1.5 mL Eppendorf tubes, and dried using a Savant SpeedVac rotary evaporator for 2 hours (SPD2010, Thermo Fisher Scientific, Waltham, MA, USA).

At the end of the drying step, 1 mL of MilliQ water is added to each sample, followed by vortexing. The samples undergo 10 minutes of sonication before being centrifuged at maximum speed and 4°C for 10 minutes.

Before analysis, samples were diluted with MilliQ water at a 50x ratio. Quantification of sucrose, fructose, and glucose is carried out using a high-performance ion chromatograph (ICS-5000, Thermo Fisher Scientific) equipped with an anion exchange CarboPac PA1 column (250 mm) at 25°C. The eluent consists of 100 nM NaOH at a flow rate of 0.25 mL/min. Detection is performed using pulsed amperometry, and chromatogram analysis, along with sugar concentration quantification, is conducted using Chromeleon software (Thermo Fisher Scientific).
